# Conserved mechanisms of self-renewal and pluripotency in mouse and human ESCs regulated by simulated microgravity using a 3D clinostat

**DOI:** 10.1038/s41420-024-01846-2

**Published:** 2024-02-09

**Authors:** Ying Ye, Wenyan Xie, Zhaoru Ma, Xuepeng Wang, Yi Wen, Xuemei Li, Hongqian Qi, Hao Wu, Jinnan An, Yan Jiang, Xinyi Lu, Guokai Chen, Shijun Hu, Elizabeth A. Blaber, Xi Chen, Lei Chang, Wensheng Zhang

**Affiliations:** 1 Medical College of Soochow University, Suzhou, China; 2 Shenzhen Key Laboratory of Gene Regulation and Systems Biology, School of Life Sciences, Southern University of Science and Technology, Shenzhen, China; 3 School of Basic Medical Sciences, Binzhou Medical University, Yantai, China; 4 State Key Laboratory of Medicinal Chemical Biology, Nankai University, Tianjin, Tianjin, 300350 China; 5 Institute of Blood and Marrow Transplantation, Medical College of Soochow University, Suzhou, China; 6 School of Biology and Basic Medical Sciences, Medical College of Soochow University, Suzhou, China; 7 Centre of Reproduction, Development and Aging, Faculty of Health Sciences, University of Macau, Taipa, Macao SAR China; 8 Department of Cardiovascular Surgery of the First Affiliated Hospital & Institute for Cardiovascular Science, Collaborative Innovation Center of Hematology, State Key Laboratory of Radiation Medicine and Protection, Medical College of Soochow University, Suzhou, 215000 China; 9 Department of Biomedical Engineering and Center for Biotechnology and Interdisciplinary Studies, Rensselaer Polytechnic Institute, Troy, NY 12180 USA; 10 State Key Laboratory of Radiation Medicine and Protection, School of Radiation Medicine and Protection, Collaborative Innovation Center of Radiation Medicine of Jiangsu Higher Education Institutions, Jiangsu Province International Joint Laboratory For Regeneration Medicine, Medical College of Soochow University, Suzhou, China

**Keywords:** Embryonic stem cells, Self-renewal, Stem-cell differentiation

## Abstract

Embryonic stem cells (ESCs) exhibit unique attributes of boundless self-renewal and pluripotency, making them invaluable for fundamental investigations and clinical endeavors. Previous examinations of microgravity effects on ESC self-renewal and differentiation have predominantly maintained a descriptive nature, constrained by limited experimental opportunities and techniques. In this investigation, we present compelling evidence derived from murine and human ESCs, demonstrating that simulated microgravity (SMG)-induced stress significantly impacts self-renewal and pluripotency through a previously unidentified conserved mechanism. Specifically, SMG induces the upregulation of heat shock protein genes, subsequently enhancing the expression of core pluripotency factors and activating the Wnt and/or LIF/STAT3 signaling pathways, thereby fostering ESC self-renewal. Notably, heightened Wnt pathway activity, facilitated by Tbx3 upregulation, prompts mesoendodermal differentiation in both murine and human ESCs under SMG conditions. Recognizing potential disparities between terrestrial SMG simulations and authentic microgravity, forthcoming space flight experiments are imperative to validate the impact of reduced gravity on ESC self-renewal and differentiation mechanisms.

## Introduction

As human exploration of outer space expands, the eventual need for prolonged stays and potential reproduction in these environments becomes inevitable. Therefore, investigating the impact of the space environment on human reproduction and development has become a crucial topic in space biology research [1–3]. The space environment is characterized by microgravity and cosmic radiation, which profoundly affect human physiology, including bone loss, muscle atrophy, decreased cardiovascular capacity, impaired immune function, delayed wound and fracture healing, among other effects [4–6]. Hence, it is plausible that factors associated with spaceflight may also influence human in utero development.

Embryonic stem cells (ESCs) are derived from the inner cell mass of preimplantation embryos [7–9]. These cells possess the remarkable ability to self-renew indefinitely and differentiate into nearly all cell types in the body, making them valuable tools for studying mammalian reproduction and development [10, 11]. Since their discovery, numerous studies have demonstrated that ESC identity is regulated by a core regulatory network composed of signaling pathways such as LIF/STAT3, Wnt, and TGF-beta, as well as pluripotency transcription factors including OCT4, SOX2, NANOG, and KLFs, protein complexes, microRNAs, and chromatin remodeling complexes [12, 13].

Oct4 is one of the key transcription factors involved in maintaining ESC identity, and its expression level is closely linked to differentiation and the establishment of naive pluripotency in ESCs [14–16]. Studies have shown that mouse ESCs and induced pluripotent stem cells (iPSCs) cultured under microgravity conditions aboard a TZ-1 space vehicle exhibited significantly higher cell survival, proliferation, and Oct4 expression compared to ground-based control groups [17, 18], suggesting that microgravity may contribute to ESC maintenance. Acharya et al. analyzed gene expression in mouse ESCs after exposure to alternating hypergravity and microgravity, revealing changes in the expression of genes involved in cell cycle regulation and cell proliferation, indicating that gravity influences ESC proliferation [19]. Recently, Timilsina et al. demonstrated increased expression of key pluripotency genes in both human ESCs and iPSCs cultured under simulated microgravity conditions [20]. Thus, accumulating evidence suggests that microgravity significantly affects the self-renewal of pluripotent stem cells (PSCs).

Due to the scarcity and high cost of flight opportunities, several microgravity devices have been developed to simulate certain aspects of spaceflight-induced microgravity conditions [21]. Unlike true microgravity experienced in space, mammalian cells cultured in simulated microgravity also encounter other physical forces such as hydrostatic pressure and fluid shear [3, 22, 23], which may lead to differences in outcomes compared to spaceflight studies. Nevertheless, multiple studies have demonstrated that both simulated and space microgravity promote the differentiation of ESCs and human iPSCs into mesodermal and mesoderm-derived cells, including contractile cardiomyocytes [1, 24–30]. Additionally, two studies found that the expression levels of endodermal markers, such as Foxa2, Sox17, and CxCr4, were significantly upregulated in differentiated ESCs under simulated microgravity by activating the Wnt pathway, indicating that simulated microgravity promotes the differentiation of mouse ESCs into endoderm [27, 31]. Another study revealed that simulated microgravity biases the differentiation of human ESCs towards caudal neural progenitor types [32]. In contrast, Timilsina et al. revealed the reduced trophectoderm and neuroectoderm differentiation of hPSCs under SMG conditions [20]. Due to technical and experimental limitations, mechanistic studies investigating the effects of microgravity on the maintenance and differentiation of ESCs remain challenging.

Heat shock proteins (HSPs) comprise a highly conserved group of proteins that are upregulated in response to stress induced by heat, as well as chemical and physical perturbations [33]. HSP90 and HSP110 (encoded by Hsph1) have been reported to regulate the growth of cancer cells through STAT3 activation [34, 35]. Here, we demonstrate that the upregulated expression of *Hsp* and *Hsf1* under simulated microgravity conditions increases the activity of LIF/STAT3 and Wnt pathways, as well as the expression of pluripotency factors *Oct4*, *Sox2*, and *Nanog* in mouse ESCs. Similar effects on the maintenance of human ESCs were observed. The increased activity of the Wnt pathway, coupled with the resulting upregulation of *Tbx3* expression, promotes the differentiation of both mouse and human ESCs into meso-endodermal lineages under simulated microgravity conditions. In conclusion, our study supports a conserved mechanism whereby microgravity may influence the growth and development of various organisms through the regulation of Hsp genes.

## Results

### SMG affects the self-renewal of mESCs

**Fig. 1:**
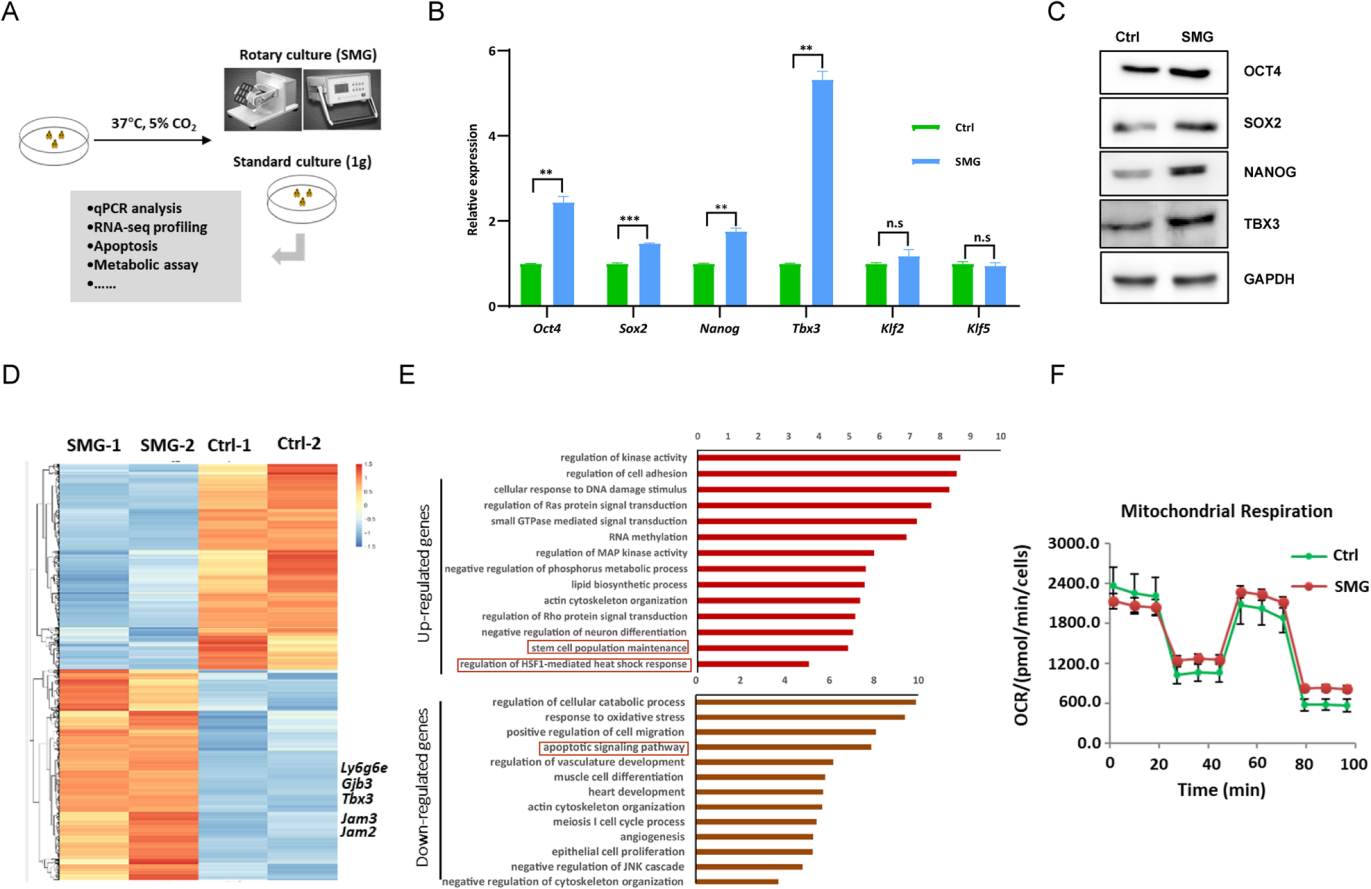
Simulated microgravity affects the self-renewal of mESCs. **A** Schematics of the experimental design. mESCs were cultured under rotary and standard conditions at 37 °C in 5% CO_2_. Cells were harvested and used for subsequent analyses. **B** Transcript levels of pluripotency-associated genes in mESCs cultured for 4 days under 1 g and SMG conditions based on qPCR analysis. (*n* = 3 independent experiments, **p < 0.01,***p < 0.001, n.s, not significant). **C** Western blot for OCT4, SOX2, NANOG and TBX3 protein levels in mESCs cultured for 4 days under 1 g and SMG conditions; GAPDH served as a loading control. (*n* = 3 independent experiments). **D** Heat map depicts the changes in gene expression in mESCs cultured under 1 g and SMG conditions for 4 days. The color represents the Z score (row-wise) of the log2 FPKM values of the 4474 genes affected. **E** GO ontology analysis for biological processes associated with genes whose expression changed under SMG condition in (**D**). **F** Oxygen consumption rate (OCR) of mESCs cultured under 1 g and SMG conditions for 4 days measured with Mito stress test. (*n* = 3 independent experiments).

To investigate the impact of SMG on the self-renewal of mESC, mESCs were cultured in a microgravity simulated Rotary Cell Culture System (RCCS, National Space Science Center, China) (Fig. 1A). The expression of core pluripotency regulators such as *Oct4*, *Sox2*, *Nanog* and *Tbx3* increased at both the transcript and protein levels (Fig. 1B, C). However, the expression of other pluripotency factors, such as *Klf2* and *Klf5* remained unchanged under SMG (Fig. 1B). To ascertain that the observed upregulation of core pluripotency genes was not a result of shear stress induced by fluid motion, we conducted an experiment involving horizontal shaking of mESCs. We subsequently examined the expression of pluripotency-related genes in mESCs cultured within flasks placed on a horizontal shaker. Interestingly, the expression levels of *Oct4*, *Sox2*, and *Tbx3* demonstrated only minimal alterations, while the expression of *Nanog* was downregulated under these shaking conditions (Fig. S1A). Collectively, these findings provide evidence that SMG governs the self-renewal of ESCs by orchestrating the expression of core pluripotency genes.

To elucidate the underlying mechanism by which SMG influences ESC self-renewal, we performed RNA sequencing (RNA-seq) to analyze global gene expression changes in ESCs under SMG. We identified 2262 significantly up- and 2212 down-regulated genes in response to SMG compared to the 1 g ground environment (Fig. 1D, Table S3). The dysregulation of genes associated with stem cell maintenance was observed, consistent with the perturbed self-renewal of ESCs under SMG (Fig. 1B, C, E). Moreover, we found enrichment of apoptosis-associated gene ontology (GO) terms among the downregulated genes in mESCs under SMG, suggesting the induction of apoptosis in mESCs (Fig. 1E). Indeed, after 96 h of culture under SMG, mESCs exhibited an increased propensity for apoptosis compared to the 1 g environment (Fig. S1B). KEGG enrichment analysis further revealed significant alterations in metabolic pathways involved in amino acid metabolism (glutathione, arginine, and proline metabolism) and carbohydrate metabolism (glyoxylate and dicarboxylate metabolism, and pyruvate metabolism) under SMG (Fig. S1C). Additionally, the mitochondrial oxygen consumption rate (OCR) of mESCs was lower under SMG, with reduced basal respiration (BR) and ATP production (AP) (Figs. 1F and S1D).

### SMG regulates LIF/STAT3 pathway in mESCs

**Fig. 2:**
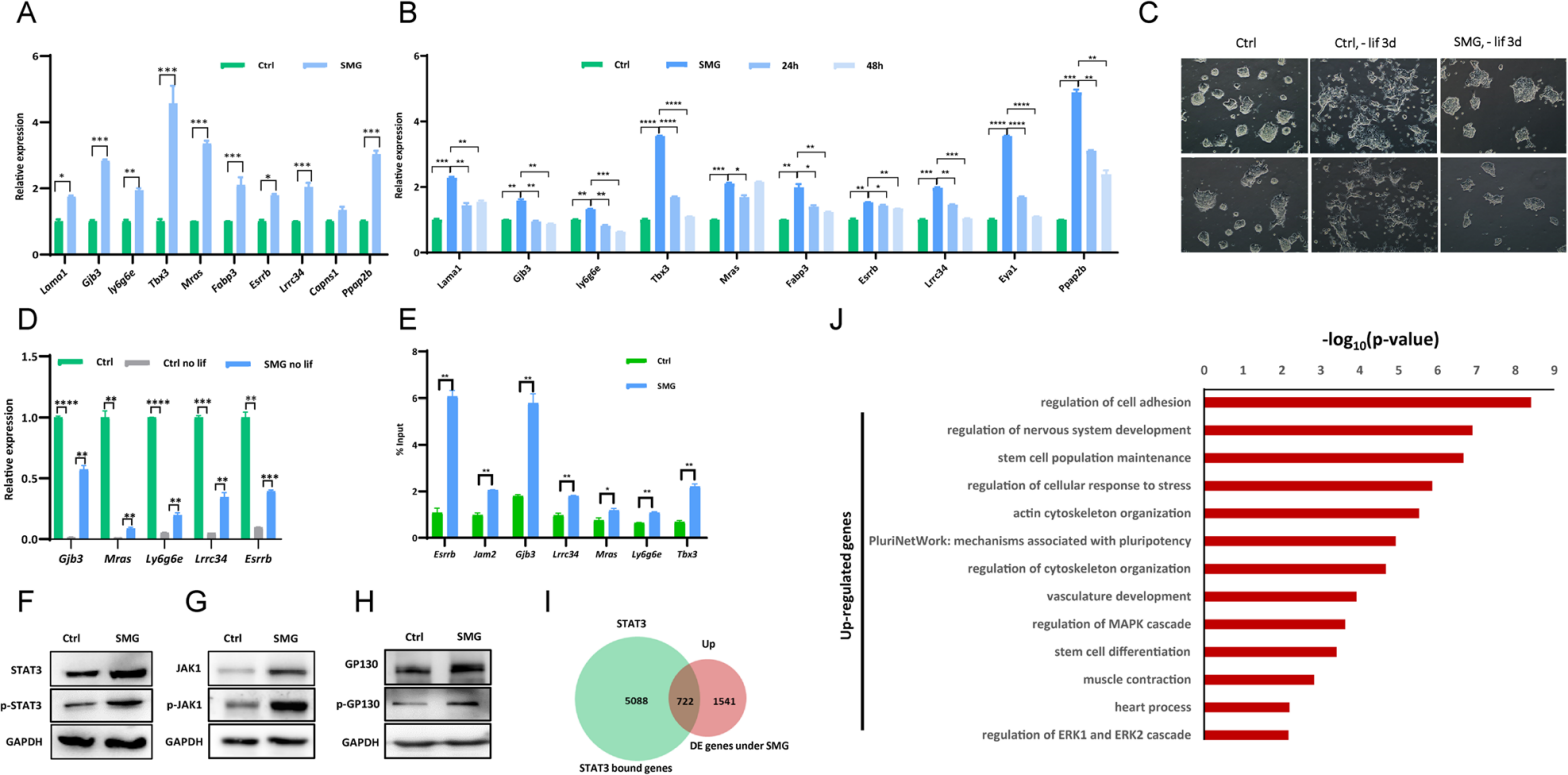
Enhanced activation of the LIF/STAT3 pathway in mESCs under SMG condition. **A** Differential expression of LIF/STAT3 target genes in mESCs after 4-day culture under SMG and 1 g conditions, assessed by qPCR analysis. The data represents 3 independent experiments (*n* = 3), with statistical significance indicated as *p < 0.05, **p < 0.01, and ***p < 0.001. **B** qPCR analysis of transcript levels of LIF/STAT3 target genes in mESCs cultured for 4 days under SMG, normal gravity (1 g), and 4 days of culture under SMG followed by 24 h or 48 h of culture under 1 g conditions. The data represents 3 independent experiments (*n* = 3), with statistical significance indicated as *p < 0.05, **p < 0.01, ***p < 0.001, and ****p < 0.0001. **C** The morphology of mESCs in ES medium with or without LIF supplement, cultured under normal gravity (1 g, Ctrl) and microgravity (SMG) conditions for 3 days. Specifically, mESCs were initially cultured under 1 g and SMG conditions for 24 h, followed by LIF withdrawal for 3 days. The data represents 3 independent experiments (*n* = 3). Scale bars represent 200 μm. **D** qPCR analysis of transcript levels of LIF/STAT3 target genes in ES medium with or without LIF, cultured under normal gravity (1 g, Ctrl) and microgravity (SMG) conditions for 3 days. The data represents 3 independent experiments (*n* = 3), with statistical significance indicated as **p < 0.01, ***p < 0.001, and ****p < 0.0001. **E** Quantification of p-STAT3 levels at LIF/STAT3 target genes in mESCs cultured under normal gravity (1 g) and microgravity (SMG) conditions, determined by ChIP-qPCR. The data represents 3 independent experiments (*n* = 3), with statistical significance indicated as *p < 0.05 and **p < 0.01. Western blot analysis of STAT3 and p-STAT3 levels (**F**), JAK1 and p-JAK1 levels (**G**), and GP130 and p-GP130 levels (**H**) in mESCs cultured for 4 days under normal gravity (1 g) and microgravity (SMG) conditions. GAPDH was used as a loading control. The data represents 3 independent experiments (*n* = 3). **I** Venn diagram illustrating the overlapping genes that are up-regulated in mESCs cultured under microgravity (SMG) conditions and bound by STAT3 protein. The diagram showcases the shared number of genes between the two conditions. **J** Gene Ontology (GO) ontology analysis revealing biological processes associated with STAT3 target genes that are upregulated under microgravity (SMG) condition. The analysis identifies the functional categories enriched among the upregulated genes.

The LIF/STAT3 pathway is essential for the maintenance of mouse ESCs [36, 37]. RNA-seq analysis revealed the upregulation of LIF/STAT3 target genes, including *Tbx3*, *Gjb3*, *Ly6g6e*, *Jam2*, and *Jam3*, in mESCs cultured for 4 days under simulated microgravity (SMG) conditions (Fig. 1D). qPCR experiments further validated the increased expression of LIF/STAT3 target genes, such as *Lama1*, *Gjb3*, *Ly6g6e*, *Tbx3*, *Mras*, *Fabp3*, *Esrrb*, *Lrrc34*, *Capns1*, and *Ppap2b*, in response to SMG (Fig. 2A). Time course experiments demonstrated sustained upregulation of LIF/STAT3 target genes in mESCs cultured for 3 days under SMG conditions (Fig. S2A). We subsequently examined the expression of LIF/STAT3 target genes in mESCs cultured within flasks placed on a horizontal shaker. The expression levels of *Tbx3*, *Gjb3*, *Lama1*, *Esrrb*, and *Ly6g6e* demonstrated minimal alterations under the shaking conditions (Fig. S2B), ascertaining the observed upregulation of LIF/STAT3 genes was not a result of shear stress induced by fluid motion. Notably, the increased expression of LIF/STAT3 target genes returned to control levels upon transferring the cells back to normal gravity for 24 and 48 h (Fig. 2B). As expected, withdrawal of LIF under SMG conditions restored the expression of LIF/STAT3 target genes (Fig. S2C). Interestingly, while LIF withdrawal resulted in differentiated ESC morphology under normal gravity, ESCs cultured in non-LIF medium under SMG conditions exhibited morphology similar to ESCs in LIF-containing medium under normal gravity (Fig. 2C). This similarity may be attributed to the higher expression of pluripotency genes *Oct4*, *Sox2*, *Nanog*, and LIF/STAT3 target genes in ESCs cultured in non-LIF medium under SMG (Figs. 2D and S2D). ChIP-qPCR experiments confirmed the enhanced enrichment of phosphorylated STAT3 (p-STAT3) at LIF/STAT3 target genes in ESCs cultured under SMG conditions (Fig. 2E). The activation of Janus kinases (JAKs) occurs through the heterodimerization of LIFR and GP130 [38, 39]. Activated JAKs phosphorylate tyrosine residues on GP130, creating a docking site for STAT3 recruitment and subsequent activation through phosphorylation at tyrosine 705 [40, 41]. Western blot analysis revealed increased levels of both STAT3 and p-STAT3 under SMG (Fig. 2F). The elevated levels of p-STAT3 may be attributed to increased levels of JAK1, phosphorylated JAK1 (p-JAK1), GP130, and phosphorylated GP130 (p-GP130) in ESCs under SMG conditions (Fig. 2G, H). BRG1, the catalytic subunit with ATPase activity of the BAF chromatin remodeling complex, is essential for facilitating STAT3 access to its target loci [42]. However, the protein level and binding of BRG1 at STAT3 target genes remained unchanged under SMG conditions (Fig. S2E, F), indicating that SMG does not regulate the binding of STAT3 to its target genes through BRG1. Consistently, deletion of Arid1a, a subunit of the BAF complex, did not affect the upregulation of LIF/STAT3 target genes under SMG (Fig. S2G). The restoration of LIF/STAT3 target gene expression back to wild-type levels at normal gravity upon Arid1a deletion suggests that SMG does not influence STAT3 binding to its target genes through the BAF complex (Fig. S2G). Therefore, we conclude that SMG enhances the activity of the LIF/STAT3 pathway in a BRG1-independent manner.

In addition to the JAK/STAT3 pathway, the binding of LIF to its receptor initiates signaling through the PI3K (phosphoinositide 3-kinase)/AKT pathway and the SHP2 domain-containing tyrosine phosphatase 2/MAPK pathway [43]. Western blot analysis revealed increased phosphorylation of both AKT and MAPK under SMG conditions, indicating that LIF binding to its receptor may be enhanced during SMG [43]. Western blots revealed the increased phosphorylation of both AKT and MAPK under SMG (Fig. S2H), supporting that the binding of LIF with its receptor may increase under SMG.

We performed a comparison between genes affected by SMG and STAT3 target genes and identified 722 upregulated STAT3 target genes under SMG conditions. These genes are associated with ESC maintenance and differentiation, cytoskeleton organization, and MAPK regulation (Fig. 2I, J). Additionally, 688 STAT3 target genes were down-regulated under SMG (Fig. S2I), and they are functionally related to the apoptotic signaling pathway, ATP metabolic process, pyruvate metabolic process, glycolytic process, and oxidative phosphorylation regulation (Fig. S2J). Given the observed increase in apoptosis and decreased ATP production in the mitochondria of ESCs under SMG (Fig. S1B, D, F), it is likely that SMG influences the maintenance, differentiation, apoptosis, and metabolism of ESCs by modulating the activity of the LIF/STAT3 pathway.

### SMG regulates LIF/STAT3 pathway via controlling the expression of heat shock protein genes

**Fig. 3:**
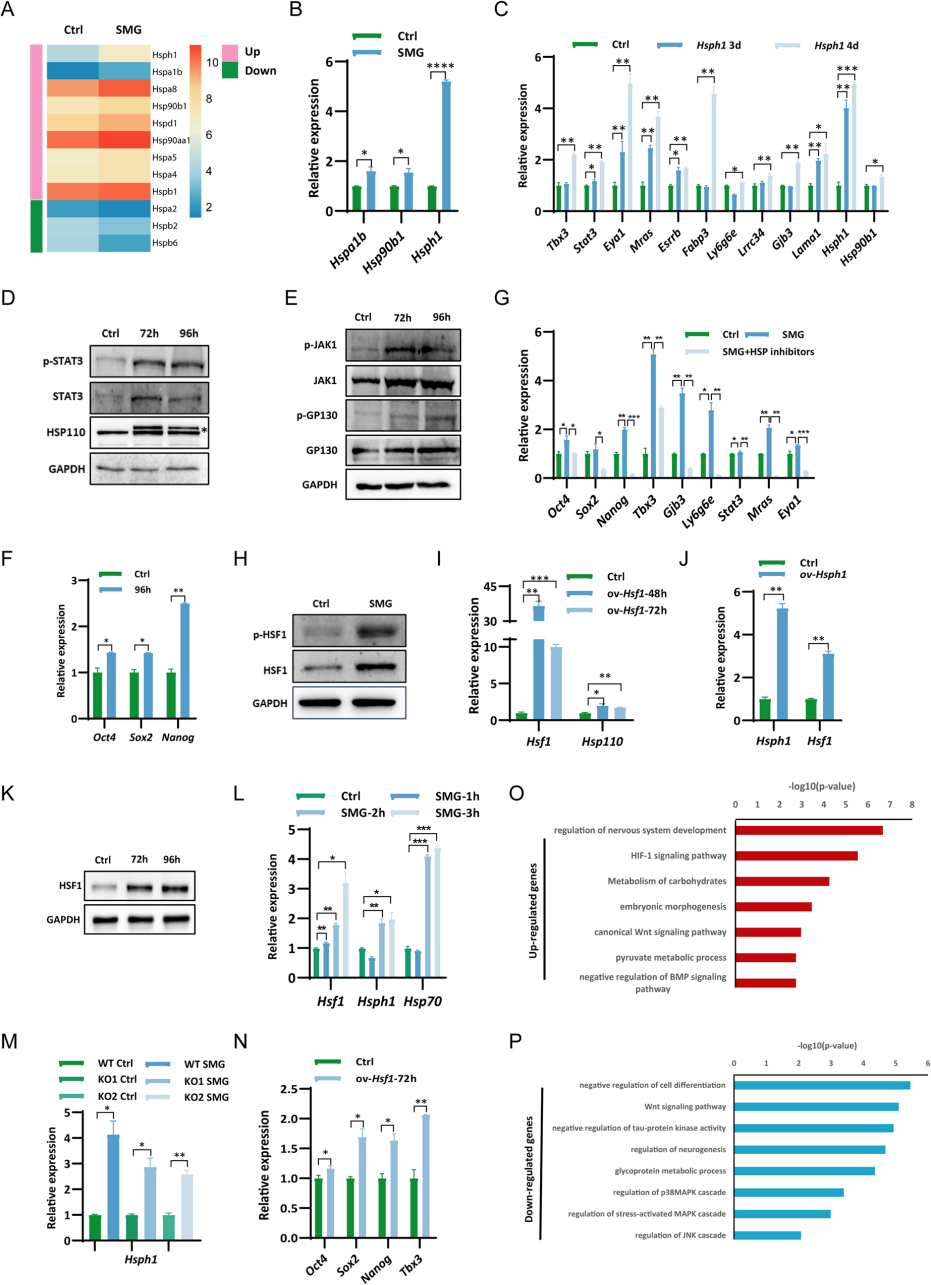
Regulation of LIF/STAT3 target genes and pluripotency genes by HSF1/HSP proteins. **A** Heat map depicting the expression changes of heat shock genes in mESCs cultured for 4 days under normal gravity (1 g) and microgravity (SMG) conditions. The color gradient represents the log2 FPKM (Fragments Per Kilobase Million) values of the genes. **B** qPCR analysis of transcript levels of *Hspa1b*, *Hsp90b1*, and *Haph1* in mESCs cultured for 4 days under normal gravity (1 g) and microgravity (SMG) conditions. The data represents 3 independent experiments (*n* = 3), with statistical significance indicated as *p < 0.05 and ****p < 0.0001. **C** qPCR analysis of transcript levels of LIF/STAT3 target genes *Tbx3*, *Stat3*, *Eya1*, *Mras*, *Esrrb*, *Fabp3*, *Ly6g6e*, *Lrrc34*, *Gjb3*, *Lama1* and *Hsp90b1* in mESCs with *Hsph1* overexpressed for 3 and 4 days, respectively. The data represents 3 independent experiments (*n* = 3), with statistical significance indicated as *p < 0.05, **p < 0.01, and ***p < 0.001. **D** Western blot analysis of STAT3 and p-STAT3 levels in mESCs with *Hsph1* overexpressed for 3 and 4 days. GAPDH was used as a loading control. The asterisk (*) indicates GFP fused HSP110. The data represents 3 independent experiments (*n* = 3). **E** Western blot analysis of JAK1 and p-JAK1, GP130 and p-GP130 levels in mESCs with *Hsph1* overexpressed for 3 and 4 days. GAPDH was used as a loading control. The data represents 3 independent experiments (*n* = 3). **F** qPCR analysis of transcript levels of pluripotency genes *Oct4*, *Sox2*, and *Nanog* in mESCs with *Hsph1* overexpressed for 4 days. The data represents 3 independent experiments (*n* = 3), with statistical significance indicated as *p < 0.05 and **p < 0.01. **G** qPCR analysis of transcript levels of indicated genes in mESCs cultured under normal gravity (1 g) and microgravity (SMG) conditions for 3.5 days. Subsequently, the cells cultured under SMG condition were treated with or without HSP inhibitors for an additional 10 h. The data represents 3 independent experiments (*n* = 3), with statistical significance indicated as *p < 0.05, **p < 0.01, and ***p < 0.001. **H** Western blot analysis of HSF1 and p-HSF1 levels in mESCs cultured for 4 days under normal gravity (1 g) and microgravity (SMG) conditions. The data represents 3 independent experiments (*n* = 3). **I** qPCR analysis of transcript levels of *Hsph1* gene in mESCs with *Hsf1* overexpressed for 2 days and 3 days. The data represents 3 independent experiments (*n* = 3), with statistical significance indicated as *p < 0.05, **p < 0.01, and ***p < 0.001. **J** qPCR analysis of transcript levels of *Hsf1* in mESCs with *Hsph1* overexpressed for 3 days. The data represents 3 independent experiments (*n* = 3), with statistical significance indicated as **p < 0.01. **K** Western blot analysis of HSF1 levels in mESCs with *Hsph1* overexpression for 72 and 96 hours, with GAPDH as a loading control. **L** qPCR analysis of transcript levels of *Hsf1*, *Hsph1* and *Hsp70* in mESCs cultured under normal gravity (1 g) and simulated microgravity (SMG) conditions for 1, 2 and 3 h. (*n* = 3 independent experiments, *p < 0.05). **M** qPCR analysis of transcript levels of *Hsph1* in WT and *Hsf1* KO mESCs cultured for 1 day under both normal gravity (1 g) and SMG conditions. (*n* = 3 independent experiments, *p < 0.05, **p < 0.01). **N** qPCR analysis of transcript levels of *Oct4*, *Sox2*, *Nanog* and *Tbx3* in mESCs with *Hsf1* overexpression for 3 days. (*n* = 3 independent experiments, *p < 0.05, **p < 0.01). GO analysis revealed the enriched GO terms of genes associated with increased (**O**) H3K27ac levels and decreased (**P**) H3K27ac levels in mESCs cultured for 11 h under simulated microgravity (SMG) and normal gravity (1 G) conditions, respectively.

GO analysis revealed that genes upregulated under SMG were associated with the regulation of heat shock factor 1 (Hsf1)-mediated heat shock response (Fig. 1E). RNA-seq analysis further demonstrated the dysregulation of HSP genes in mESCs under SMG conditions (Fig. 3A). Intriguingly, the expression of hsp genes in barley and Arabidopsis has also been reported to be altered in the space environment [44, 45]. Considering that HSP90 and HSP110 have been implicated in regulating the activity of STAT3 [34, 35], we hypothesized that SMG might modulate the activity of the LIF/STAT3 pathway by influencing the expression of Hsp genes. qPCR experiments confirmed the increased expression of *Hspa1b*, *Hsp90b1*, and *Hsph1* in mESCs under SMG (Fig. 3B), which was restored upon transfer to normal gravity for 24 h (Fig. S3A). Notably, *Hsph1* exhibited the most significant alteration in expression under SMG conditions (Fig. 3A, Table S3). To investigate the impact of Hsph1 on the LIF/STAT3 pathway, we overexpressed *Hsph1* in mESCs and examined its effects. Overexpression of *Hsph1* resulted in elevated mRNA levels of *Stat3* and its target genes (Fig. 3C). Moreover, protein analysis revealed increased levels of STAT3, p-STAT3, JAK1, p-JAK1, GP130, and p-GP130 in mESCs upon Hsph1 overexpression (Fig. 3D, E), providing support for the notion that *Hsph1* overexpression enhances the activity of the LIF/STAT3 pathway. Consistent with the increased expression of pluripotency genes observed under SMG conditions (Fig. 1B, C), *Hsph1* overexpression also upregulated the expression of *Oct4*, *Sox2*, *Nanog*, and *Tbx3* (Figs. 3C, F, S3B). These findings suggest that SMG may regulate the expression of pluripotency genes by modulating *Hsph1* expression.

In addition to Hsph1, the upregulation of *Hsp90b1* was also observed under SMG conditions (Figs. 3B, S3A). Similar to *Hsph1*, overexpression of *Hsp90b1* resulted in increased expression of *Stat3*, STAT3 target genes, and pluripotency genes *Oct4*, *Sox2*, and *Nanog* (Fig. S3C, D). Notably, the co-overexpression of *Hsph1* and *Hsp90b1* further enhanced the expression of STAT3 target genes, including *Tbx3*, *Eya1*, and *Mras* (Fig. S3C), suggesting a collaborative regulation of the LIF/STAT3 pathway by different Hsp genes. The upregulation of other Hsp genes such as *Hspa8*, *Hsp90aa1*, and *Hspb1* under SMG conditions indicates their potential contribution to the upregulation of pluripotency genes and LIF/STAT3 target genes (Fig. 3A). To further confirm the hypothesis that the upregulation of pluripotency genes and LIF/STAT3 target genes under SMG conditions is mediated by Hsp genes, we employed inhibitors to block the induction of HSP proteins, including HSP inhibitor I (HSP70 and HSP105 inhibitor) and 17-AAG (HSP90 inhibitor). The addition of HSP inhibitors to ESCs cultured under SMG conditions significantly impaired the upregulation of pluripotency genes *Oct4*, *Sox2*, *Nanog*, *Stat3*, and LIF/STAT3 target genes such as *Tbx3*, *Gjb3*, *Ly6g6e*, *Mras*, and *Eya1* (Fig. 3G). This experimental evidence supports our conclusion that the induction of Hsp genes under SMG conditions leads to the upregulation of pluripotency genes and LIF/STAT3 target genes in ESCs.

HSF1, a key regulator of the heat shock response [46], was hypothesized to be involved in the regulation of hsp genes under SMG conditions. Indeed, we observed an increase in the expression of Hsf1 and its active form p-HSP1 at both the mRNA and protein levels under SMG (Figs. 3H and S3E). Overexpression of Hsf1 resulted in the upregulation of Hsph1 (Fig. 3I), while overexpression of Hsph1 upregulated Hsf1 expression (Fig. 3J, K). Notably, as early as 2 h of culture under SMG, we observed the upregulation of *Hsph1*, *Hsf1*, and *Hsp70* in mESCs (Fig. 3L). These findings suggest that Hsph1 and Hsf1 may be independently upregulated under SMG, while also co-regulating each other. To further investigate this, we employed CRISPR/Cas9 technology to generate *Hsph1* and *Hsf1* knockout ESCs, and Western blot analysis confirmed the successful deletion of *Hsph1* and *Hsf1* genes (Fig. S3F–G). Importantly, the deletion of either *Hsph1* or *Hsf1* did not affect the upregulation of the other gene under SMG conditions (Figs. 3M and S3H), providing further support for the independent regulation of Hsph1 and Hsf1 under SMG. Overexpression of *Hsf1* led to an increased expression of pluripotency genes *Oct4*, *Sox2*, *Nanog*, and *Tbx3* (Fig. 3N), as well as *Stat3* and LIF/STAT3 target genes (Fig. S3I). Addition of HSP inhibitors in *Hsf1* KO ESCs cultured under SMG conditions significantly decreased the expression of pluripotency genes *Oct4*, *Sox2*, *Nanog*, and LIF/STAT3 target genes *Tbx3*, *Gjb3*, *Ly6g6e*, *Mras*, *Lama1*, and *Eya1* (Fig. S3J). Importantly, compared to WT ESCs, the addition of HSP protein inhibitors to Hsf1 KO ESCs cultured under SMG resulted in a greater decrease in the expression of the upregulated pluripotency and LIF/STAT3 target genes (Figs. 3G and S3J, K), further supporting the independent regulation of Hsf1 and Hsp genes in the context of pluripotency and STAT3 target gene regulation.

Microgravity-induced transcriptional alterations have been found to be regulated at the epigenetic level as an adaptive response to the space microgravity environment [6, 47]. To investigate the role of histone modifications in the regulation of gene expression under SMG conditions, we conducted genome-wide profiling of H3K4me3, H3K27ac, and H3K27me3 in mESCs cultured under SMG for 11 h. Differential binding analysis revealed 1331 regions with increased H3K4me3 levels and 190 regions with decreased H3K4me3 levels (Fig. S3L, left). Similarly, 1307 regions showed increased H3K27ac levels, while 696 regions showed decreased H3K27ac levels (Fig. S3L, middle). No significant changes were observed in H3K27me3 levels under SMG conditions (Fig. S3L, right). To further characterize the affected genomic regions, we employed a hidden Markov model (ChromHMM) to classify the genome into distinct segments based on the histone ChIP-seq data from mESCs [48]. A total of 15 states representing different types of promoters, enhancers, and other genomic regions were identified [48]. Analysis of the differential H3K27ac peaks between 1 g and SMG environments revealed their predominant localization in promoter and enhancer regions (Fig. S3M), suggesting the regulation of gene activity through control of promoters and enhancers under SMG conditions. Gene ontology (GO) analysis revealed that genes associated with increased H3K27ac levels were functionally related to HIF-1 and Wnt signaling pathways, as well as metabolism (Fig. 3O). In contrast, genes with decreased H3K27ac levels were associated with cell proliferation, stress-activated MAPK, and JNK regulation (Fig. 3P). Furthermore, genes associated with increased and decreased H3K4me3 modifications were related to cell fate commitment and microtubule depolymerization, respectively (Fig. S3N–O). Consequently, the GO analysis of genes linked to differential H3K27ac and H3K4me3 peaks was consistent with the dysregulated genes identified from RNA-seq data under SMG conditions (Figs. 3O, P and S3N, O, Fig. 1E), providing further support for the regulation of gene expression under SMG conditions through the control of histone modifications.

### The promotion of mesoendoderm differentiation in mESCs under SMG environment was achieved by controlling the expression of Tbx3

**Fig. 4:**
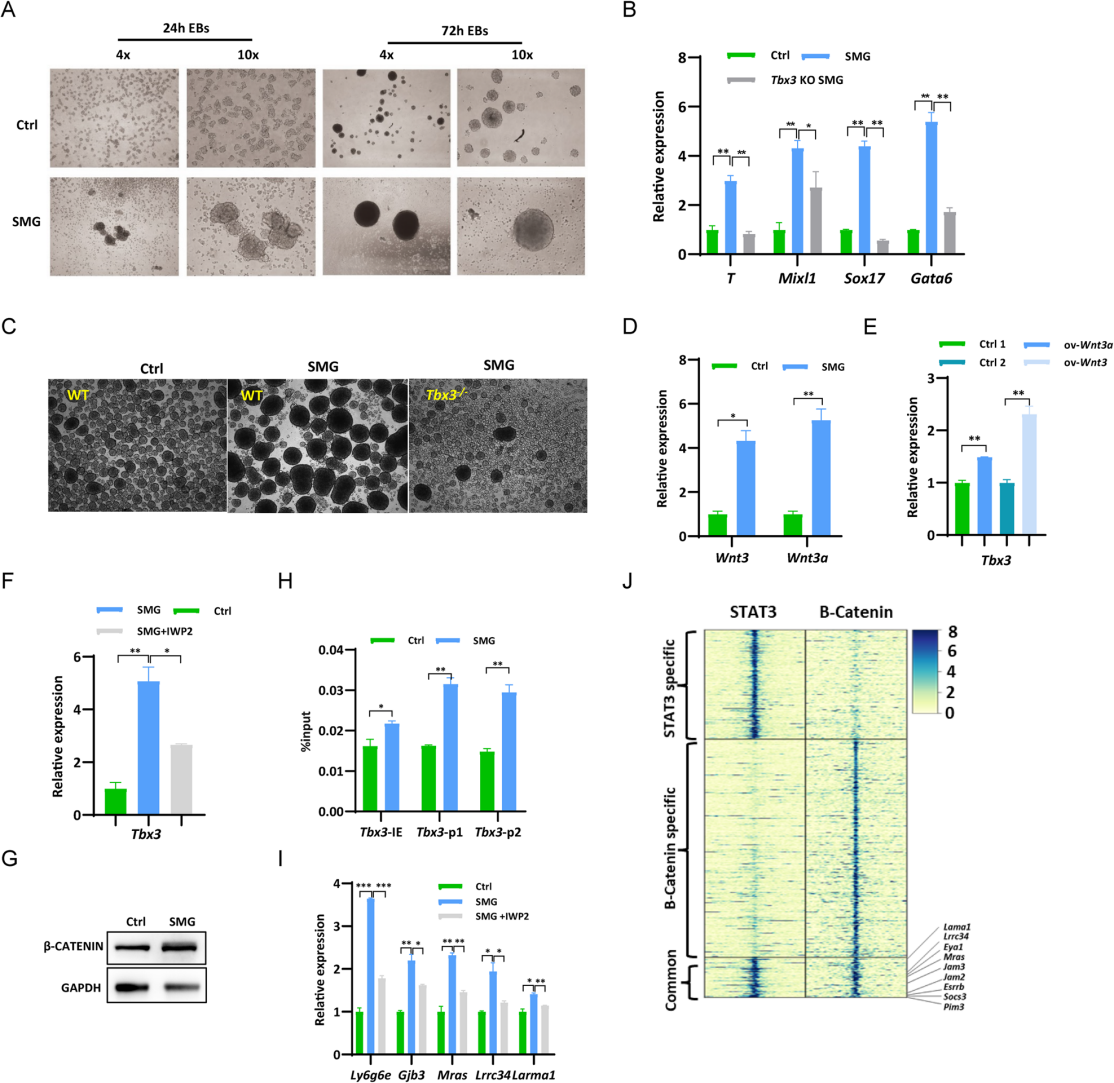
SMG promotes mesoendoderm differentiation of mESC by regulating the Wnt signaling pathway and Tbx3 Expression. **A** Morphology of embryoid bodies (EBs) induced under 1 g and SMG conditions at the indicated time points. Scale bars, 500 μm (4×), 200 μm (10x). **B** qPCR analysis of transcript levels of mesoderm markers *T* and *Mixl1*, and endoderm markers *Sox17* and *Gata6* in EBs induced under 1 g and SMG conditions on day 3. (*n* = 3 independent experiments, *p < 0.05, **p < 0.01). **C** Morphology of 3-day EBs induced from WT and *Tbx3* KO mESCs under 1 g and SMG conditions. Scale bars, 500 μm. **D** qPCR analysis of transcript levels of *Wnt3* and *Wnt3a* in mESCs under 1 g and SMG conditions for 4 days. (*n* = 3 independent experiments, *p < 0.05, **p < 0.01). **E** qPCR analysis of Tbx3 expression in WT and mESCs with overexpression of Wnt3 or Wnt3a. (*n* = 3 independent experiments, **p < 0.01). **F** qPCR analysis of transcript levels of *Tbx3* gene in mESCs under SMG condition supplemented with or without 10 μg/ml of IWP2 for 4 days. (*n* = 3 independent experiments, *p < 0.05, **p < 0.01). **G** Western blot for β-CATENIN levels in mESCs cultured for 4 days under 1 g and SMG conditions, with GAPDH as the loading control. (*n* = 3 independent experiments). **H** β-CATENIN levels at promoter and intronic enhancer regions of *Tbx3* gene in mESCs cultured for 4 days under 1 g and SMG conditions determined by ChIP-qPCR. (*n* = 3 independent experiments, *p < 0.05, **p < 0.01). **I** qPCR analysis of transcript levels of LIF/STAT3 target genes Ly6g6e, Gjb3, Mras, Lrrc34, and Lama1 in mESCs cultured under SMG conditions with and without 10 μg/ml of IWP-2 for 4 days (*n* = 3 independent experiments, *p < 0.05, **p < 0.01, ***p < 0.001). **J** The heat map displays ChIP-seq signals of STAT3 and β-CATENIN surrounding specific binding sites for STAT3, β-CATENIN, and their shared binding sites. The color gradient represents the log2 RPM (Reads Per Million) values.

To investigate the differentiation potential of mESCs under SMG environment, we conducted embryoid body (EB) differentiation assays. EBs generated under SMG exhibited increased size and density compared to those formed under normal gravity (1 g) conditions (Fig. 4A), indicating a significant impact of SMG on mESC differentiation. Additionally, we examined the expression levels of established lineage markers. The expression of mesoderm markers *T* and *Mixl1*, as well as endoderm markers *Sox17* and *Gata6*, was significantly upregulated in EBs induced under SMG (Fig. 4B). Previous studies, including our own, have demonstrated that Tbx3 plays a crucial role in regulating mesoendoderm differentiation of mESCs [48–50]. Notably, *Tbx3* expression was found to be increased under SMG conditions (Fig. 1B, C). Based on these findings, we hypothesized that the elevated expression of Tbx3 under SMG might contribute to the preferential differentiation of mESCs towards mesoendoderm lineages. To test this hypothesis, we induced the formation of EBs using *Tbx3* knockout (KO) mESCs [48]. The expression levels of mesoendoderm marker genes *T*, *Mixl1*, *Sox17*, and *Gata6* in *Tbx3* KO EBs induced under SMG were comparable to those observed in wild-type (WT) EBs under 1 g gravity control conditions (Fig. 4B). Moreover, the size of *Tbx3* KO EBs was smaller than that of WT EBs under SMG (Fig. 4C). These findings support our conclusion that the differentiation of mESCs towards mesoendoderm lineages under SMG is partly regulated by Tbx3 expression.

A previous study by Lei et al. [27] demonstrated that mesoendoderm differentiation of mESCs can be enhanced through modulation of the Wnt/β-catenin pathway under SMG conditions. In our investigation, we observed a significant upregulation of both Wnt3 and Wnt3a under the SMG environment (Fig. 4D). Moreover, overexpression of *Wnt3* or *Wnt3a* resulted in increased expression of Tbx3 (Fig. 4E). To further elucidate the role of the Wnt pathway in Tbx3 regulation, we employed IWP2, an inhibitor of the Wnt pathway, which successfully restored the upregulated expression of *Tbx3* under SMG conditions (Fig. 4F). These findings suggest that the activated Wnt/β-catenin pathway under SMG conditions may exert control over mesoendoderm differentiation through regulation of *Tbx3* expression. β-catenin, a key effector of the Wnt signaling pathway [51], exhibited an increased protein level under the SMG environment (Fig. 4G). Importantly, our ChIP-qPCR experiments demonstrated augmented binding of β-catenin protein at the intronic enhancer and promoter regions of the *Tbx3* gene under the SMG environment [48] (Fig. 4H). Therefore, our findings further support the conclusion that SMG-induced activation of the Wnt pathway upregulates *Tbx3* expression, thereby promoting the mesoendoderm differentiation of mESCs.

### Enhanced expression of LIF/STAT3 Target genes and pluripotency factors via activation of the Wnt pathway under SMG conditions

The expression levels of LIF/STAT3 target genes were found to be upregulated under the conditions of SMG (Fig. 2A–C). In addition to Tbx3, we observed that inhibiting the Wnt pathway using IWP2 in mESCs cultured under SMG restored the expression of other LIF/STAT3 target genes, including *Ly6g6e*, *Gjb3*, *Mras*, *Lrrc34*, and *Lama1*, to levels comparable to those in mESCs under normal gravity conditions (Fig. 4I). This finding suggests the involvement of the Wnt signaling pathway in the regulation of LIF/STAT3 target genes. Further analysis through STAT3 and β-CATENIN ChIP-seq demonstrated their co-binding at LIF/STAT3 target genes such as *Lama1*, *Lrrc34*, *Eya1*, *Mras*, *Jam2/3*, *Esrrb*, *Socs3*, and *Pim3* (Fig. 4J), providing additional support for the regulatory role of the Wnt signaling pathway in LIF/STAT3 gene expression. Moreover, overexpression of *Wnt3* or *Wnt3a* resulted in increased expression of LIF/STAT3 target genes (Fig. S4A, B). We identified 2100 genomic regions co-occupied by β-catenin and STAT3 (Figs. 4J and S4C), with many of these regions overlapping with LIF/STAT3 target genes (Fig. 4J), further supporting the role of the Wnt signaling pathway in regulating LIF/STAT3 gene expression. GO analysis revealed that β-CATENIN and STAT3 co-bound sites are located proximal to genes associated with stem cell maintenance, embryonic development, and cell fate commitment (Fig. S4D). Consistent with previous findings regarding Nanog regulation by Wnt3a [52], overexpression of either *Wnt3a* or *Wnt3* increased the expression of *Oct4*, *Sox2*, and *Nanog* (Fig. S4E, F). ChIP-seq analysis of β-CATENIN revealed its binding to the *Oct4*, *Sox2*, and *Nanog* genes (Fig. S4G), indicating direct regulation of core pluripotency genes by the Wnt pathway. Overexpression of either *Hsph1* or *Hsf1* resulted in upregulation of *Wnt3* and *Wnt3a* (Fig. S4G–I), suggesting that the increased expression of *Hsf1* and *Hsp* genes under SMG conditions may lead to enhanced activity of the Wnt pathway (Fig. 4D). Therefore, our findings support the conclusion that SMG-induced expression of *Hsf1* and *Hsp* genes increases Wnt pathway activity, thereby promoting the self-renewal of mESCs under ESC maintaining conditions.

### SMG enhanced mesendoderm differentiation of human ESCs (hESCs) by controlling WNT pathway activity

**Fig. 5:**
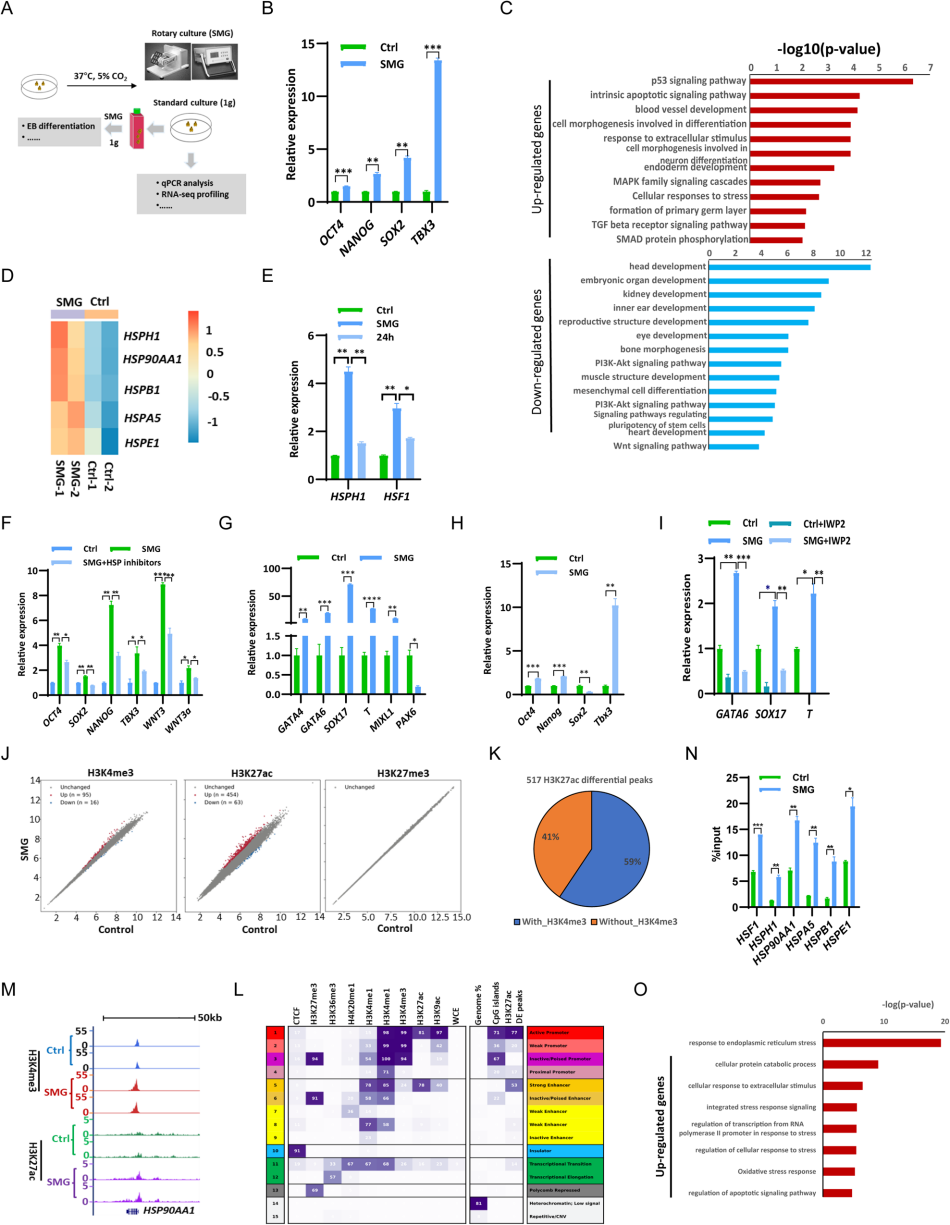
SMG affects the self-renewal and differentiation of hESCs. **A** Schematics of the experimental design. hESCs were cultured under rotary and standard conditions at 37 °C in 5% CO_2_. Cells were harvested and used for subsequent analyses. **B** qPCR analysis of transcript levels of pluripotency genes *OCT4*, SOX2, *NANOG* and *TBX3* in hESCs cultured under 1 g and SMG conditions for 4 days. (*n* = 3 independent experiments, **p < 0.01, ***p < 0.001). **C** GO ontology analysis for biological processes associated with the up- and down-regulated genes in hESCs cultured under SMG conditions. **D** The heat map illustrates the expression changes of the indicated heat shock genes in hESCs cultured under both 1 g and SMG conditions for a duration of 4 days. The color gradient represents the Z score (row-wise) of the log2 FPKM (Fragments Per Kilobase Million) values of the genes. **E** qPCR analysis of transcript levels of *HSPH1* and *HSF1* in hESCs cultured under SMG and normal gravity (1 g) conditions for a duration of 4 days, as well as 24 h of culture at 1 g following 3 days of SMG. (*n* = 3independent experiments, *p < 0.05, **p < 0.01). **F** qPCR analysis of transcript levels of indicated genes in hESCs cultured under 1 g and SMG conditions for 3 days. Subsequently, hESCs cultured under SMG condition were treated with or without HSP inhibitors for 5 h before RNA extraction for further qPCR analysis. (*n* = 3 independent experiments, *p < 0.05,**p < 0.01, ***p < 0.001). **G** qPCR analysis of transcript levels of endoderm markers *GATA4*, *GATA6*, and *SOX17*, mesoderm markers *T* and *MIXL1*, and neuronal ectoderm marker *PAX6* in day 2 EBs induced under both 1 g and SMG conditions. (*n* = 3 independent experiments, *p < 0.05,**p < 0.01, ***p < 0.001, ****p < 0.0001). **H** qPCR analysis of transcript levels of pluripotency genes *OCT4*, *SOX2*, *NANOG* and *TBX3* in day 2 EBs induced under 1 g and SMG conditions. (*n* = 3 independent experiments, **p < 0.01, ***p < 0.001). **I** qPCR analysis of transcript levels of endoderm marks *GATA6* and *SOX17*, mesoderm markers *T* in day 2 EBs induced under 1 g and SMG conditions, with and without the presence of 10 μg/ml of IWP2. (*n* = 3 independent experiments, *p < 0.05,**p < 0.01, ***p < 0.001). **J** Scatter plot showing the log RPKM values of H3K4me3 (left), H3K27ac (middle) and H3K27me3 (right) in hESCs. Significant differential peaks (|log2FC | >1 and *q* value < 0.01 by DESeq2) are shown in red (up) and blue (down) colors. **K** Overlap Percentage of Differential H3K27ac and H3K4me3 Peaks. **L** Chromatin states defined by ChromHMM using the ChIP-seq data of the histone marks. Heatmap representation of the emission probability of each histone modifications (left) and the enrichment of different genomic segments (right). **M** Genome browser view of H3K4me3 and H3K27ac at the *HSP90AA1* locus in hESCs. **N** ChIP-qPCR analyses of the *HSF1*, *HSPH1*, *HSP90AA1*, *HSPA5*, *HSPB1*, and *HAPE1* loci from hESCs cultured under 1 g and SMG conditions for 2 days were carried out with H3K27ac antibody. **O** Enriched Gene Ontology (GO) terms of genes associated with up-regulated H3K27ac in hESCs cultured under SMG and 1 g conditions.

We conducted further investigations into the effects of SMG on human embryonic stem cell (hESC) self-renewal. For this purpose, hESCs were cultured under SMG conditions using the RCCS system (Fig. 5A). qPCR analysis revealed a significant increase in the expression of core pluripotency regulators *OCT4*, *SOX2*, *NANOG*, and *TBX3* after 4 days of culture under SMG conditions (Fig. 5B). These increased expression levels returned to control levels after 24 h of culture under 1 g conditions (Fig. S5A).

To investigate the underlying mechanism of SMG-induced effects on hESC self-renewal, we performed RNA-seq to analyze global gene expression changes in hESCs under SMG. We identified 764 significantly upregulated and 1187 downregulated genes under SMG compared to the 1 g condition (Fig. S5B, Table S4). SMG led to dysregulation of genes associated with pathways involved in hESC maintenance, such as TGF-beta and Wnt pathways (Fig. 5C), which is consistent with the altered expression of core pluripotency factors (Fig. 5B). Similar to mESCs (Fig. 1E), many affected genes in hESCs were related to apoptosis and the MAPK pathway (Fig. 5C). The expression of human heat shock genes *HSPH1*, *HSP90AA1*, *HSPB1*, *HSPA5*, and *HSPE1* was upregulated under SMG for 4 days (Figs. 5D and S5B), but returned to control levels under 1 g conditions (Figs. 5E and S5C). Similar to mESCs, hESCs cultured under SMG conditions displayed upregulated expression of *HSP* gene *HSF1*, *HSPH1*, *HSP90AA1*, *HSPA5*, and *HSPB1*, even as early as 1 to 3 h of exposure (Fig. S5D). Overexpression of human HSPH1 resulted in increased expression of SOX2, NANOG, and WNT3 (Fig. S5E), suggesting that SMG may regulate the expression of these genes by controlling HSP gene expression. Consistently, the addition of HSP inhibitors to hESCs cultured under SMG conditions significantly decreased the upregulated expression of pluripotency genes *OCT4*, *SOX2*, *NANOG*, *WNT3*, and *WNT3a* (Fig. 5F). These findings demonstrate that, similar to mouse ESCs (Fig. 3G), the induction of HSP genes under SMG conditions upregulates the expression of pluripotency genes and the Wnt pathway in hESCs.

GO analysis revealed that the differentially expressed genes were associated with cell differentiation and embryonic development (Fig. 5C), indicating that the differentiation potential of hESCs may be altered under the SMG environment. To validate this observation, we performed an EB assay and examined the expression of representative marker genes from the three germ layers in day 2 and day 4 EBs induced under SMG. The expression of mesoderm markers *T* and *MIXL1*, as well as endoderm markers *GATA4*, *GATA6*, and *SOX17*, was significantly increased in EBs induced under SMG (Figs. 5G and S5E). However, the expression of the neuronal ectoderm marker gene PAX6 was lower in EBs under SMG (Figs. 5G and S5F). Therefore, SMG enhanced the differentiation of hESCs towards mesoendoderm, but impaired differentiation towards neuronal ectoderm.

To investigate the mechanism underlying the enhanced differentiation of hESCs towards mesoendoderm under SMG, we examined the expression levels of pluripotency factors *OCT4*, *SOX2*, *NANOG*, and *TBX3* in EBs. qPCR analysis revealed that the expressions of *OCT4*, *NANOG*, and *TBX3* were upregulated, with *TBX3* exhibiting the highest increase, in both day 2 and day 4 EBs induced under the SMG environment (Figs. 5H and S5G). However, the expression of *SOX2* decreased (Figs. 5H and S5F), which may explain the observed impairment in neuronal ectoderm differentiation (Figs. 5G and S5E). Furthermore, the expressions of *WNT3* and *WNT3a* were elevated in both day 2 and day 4 EBs induced under SMG (Fig. S5H), suggesting their involvement in the regulation of *TBX3* expression. To verify whether the increased activity of the WNT pathway contributed to the biased mesoendoderm differentiation of hESCs under SMG, we added the WNT pathway inhibitor IWP2 during EB induction. The inhibition of the WNT pathway by IWP2 restored the elevated expression of endoderm marker genes *GATA6* and *SOX17*, as well as the mesoderm marker gene *T*, in EBs induced under SMG conditions (Fig. 5I). Additionally, the upregulated expression of *TBX3* in day 2 EBs induced under SMG was restored to its control level in the presence of the WNT inhibitor IWP2 (Fig. S5I). Based on these findings, we conclude that the biased mesoendoderm differentiation of hESCs under SMG may be achieved by increasing the activity of the WNT pathway and subsequently upregulating the expression of *TBX3*.

HSP genes were found to be upregulated as early as 3 h under SMG conditions (Fig. S5J). To investigate the role of histone modifications in the regulation of gene expression under SMG, we analyzed the genome-wide profiles of H3K4me3, H3K27ac, and H3K27me3 in hESCs cultured under SMG for 5 h. Differential binding analysis revealed that only a small fraction of H3K4me3 peaks were altered, with 95 regions showing increased levels and 16 regions showing decreased levels of H3K4me3 (Fig. 5J, left). In contrast, a larger number of regions showed increased H3K27ac modification, with 454 regions exhibiting increased levels and 63 regions showing decreased levels of H3K27ac (Fig. 5J, Middle; Fig. S5K). Notably, 59% of the 517 differentially acetylated H3K27 regions overlapped with the H3K4me3 regions (Fig. 5K), while the remaining 41% of the H3K27ac differential peaks were located in regions without H3K4me3 (Fig. 5L). No differential H3K27me3 signals were observed (Fig. 5J, Right). ChromHMM analysis revealed that the differentially acetylated H3K27 peaks between the 1 g and SMG environments were primarily located in promoter and enhancer regions (Fig. 5L). Therefore, gene expression regulation under SMG conditions mainly occurs through the control of promoter and enhancer activity. Consistently, H3K4me3 and H3K27ac modifications increased in heat shock genes *HSP90AA1* and *HSPH1* (Figs. 5M and S5L). ChIP-qPCR analysis confirmed the augmented H3K27ac and H3K4me3 modifications in heat shock genes *HSF1*, *HSPH1*, *HSP90AA1*, *HSPA5*, *HSPB1*, and *HAPE1* in hESCs cultured under SMG conditions for 2 days (Figs. 5N and S5M). Gene ontology analysis showed that genes associated with the increased H3K27ac and H3K4me3 peaks were functionally related to stress, regulation of HSF1-mediated heat shock response, and the apoptosis signaling pathway (Figs. 5O and S5N), while genes associated with the decreased H3K27ac peaks were functionally associated with glycoprotein metabolic processes, regulation of actin cytoskeleton, and Erk1/2 regulation (Fig. S5O). Therefore, under SMG conditions, there is an initial upregulation of genes related to stress by modulating their promoter and enhancer activity in hESCs, followed by regulation of ESC maintenance and differentiation.

## Discussion

The LIF/STAT3 and Wnt signaling pathways are acknowledged as pivotal regulators in the maintenance of mESCs [36, 53]. Previous studies have highlighted their collaborative role in sustaining mESC self-renewal [54, 55]. Essential factors in the core pluripotency circuitry, Oct4, Sox2, and Nanog, play a key role in maintaining ESC self-renewal [36, 56]. Tbx3, a transcription factor in the outer core circuitry supporting self-renewal, has been shown to impair ESC self-renewal upon its depletion [49, 57]. In our study, we demonstrated that both the LIF/STAT3 and Wnt pathways were activated in mESCs cultured under SMG conditions. It should be noted that the LIF/STAT3 pathway is not essential for maintaining hESCs [58]. However, we observed an increase in the activity of the Wnt pathway in hESCs maintained under the SMG environment. Furthermore, we revealed an upregulation of pluripotency genes *Oct4*, *Nanog*, *Sox2*, and *Tbx3* in both mouse and human ESCs. This finding is consistent with a study by Timilsina et al., which reported increased expression of *OCT4*, *SOX2*, and *NANOG* in hPSCs [20]. Additionally, the expression of the core pluripotency factor Oct4 was significantly higher in both mouse and human ESCs and induced pluripotent stem cells (iPSCs) cultured in a space microgravity environment compared to ground-based controls [17, 18]. These observations suggest that the results obtained from SMG experiments align with the conditions experienced in real space environments. Consequently, both SMG and true space microgravity may contribute to the enhancement of ESC maintenance by collaboratively modulating key signaling pathways and the expression of essential pluripotency genes in ESCs. To validate these findings, conducting an RNA-seq experiment with ESCs cultured under space microgravity conditions and comparing their gene expression profiles with those obtained in our study would be necessary when future opportunities arise.

Furthermore, we demonstrated that the increased activity of the Wnt pathway in ESCs under the SMG environment resulted in the upregulation of Tbx3 expression, which, in turn, promoted mesoendoderm differentiation (Figs. 4–5). Inhibition of the Wnt pathway downregulated *Tbx3* expression and restored the elevated expression of mesoendoderm marker genes in SMG-induced EBs (Figs. 4F, 5I and S5I). Literature supports Tbx3’s role in ESC differentiation into meso-endoderm lineages [48–50]. Deleting *Tbx3* counteracted the enhanced mesoendoderm differentiation of ESCs under SMG conditions (Fig. 4A–C). Consequently, the elevated Tbx3 expression under SMG conditions contributes to both promoted self-renewal and mesoendoderm differentiation in ESCs, potentially through collaborative interactions with distinct factors. Notably, Knudsen et al. [59] reported the bipartite function of ESRRB on the self-renewal and PrE differentiation. Future investigations into these interactions would be intriguing.

The Wnt pathway is known to play a vital role in maintaining the balance between self-renewal and differentiation in various stem cell populations [60, 61]. A recent study by Cheng et al. demonstrated that SMG hinders dermal fibroblastic differentiation of BMSCs by suppressing the Wnt signaling pathway [61]. In consistent, the inhibition of Wnt pathway restored the SMG-promoted mesoendoderm differentiation of ESCs (Fig. 5I). This finding raises intriguing possibilities regarding the modulation of Wnt pathway activity by SMG and its involvement in stem cell differentiation. Further investigations could explore whether such regulatory mechanisms are conserved in other types of stem cells. Multiple studies have reported that both simulated microgravity and real space microgravity promote the differentiation of ESCs and hiPSCs into mesoderm and mesoderm-derived cell lineages [17, 25–31]. Gambacurta et al. [62] observed higher expression of endoderm markers *GATA4* and *SOX17* during osteoblastic differentiation of human iPSCs under microgravity conditions in the International Space Station (ISS) for 72 h, indicating a biased endoderm differentiation of human PSCs under space microgravity. Therefore, it is plausible that the differentiation of ESCs into mesoendoderm may be promoted under real space microgravity conditions, although this hypothesis needs to be confirmed through experiments conducted in space.Top of Form

HSPs are molecular chaperones that play a crucial role in maintaining protein homeostasis and promoting cell survival under stressful conditions [33]. The heat shock transcription factor 1 (HSF1) is the primary regulator of HSPs, binding to heat shock elements (HSEs) in the promoters of Hsp genes and orchestrating their rapid induction in response to environmental stresses [63]. In our study, we have uncovered a mechanism by which SMG enhances the maintenance of ESCs. The upregulation of Hsp genes under SMG conditions leads to increased expression of core pluripotency genes and activation of major signaling pathways in ESCs. Recent studies have also reported increased expression of HSPs in *Caenorhabditis elegans* and human endothelial cells cultured under simulated microgravity, suggesting a protective response against microgravity-related stimuli [64, 65]. Transcriptomic analysis of barley grown aboard the International Space Station (ISS) has revealed enhanced expression of HSP genes compared to ground controls [44]. Similarly, Arabidopsis cells cultured under spaceflight conditions exhibited a more than 5-fold increase in the expression of *Hsp101*, *Hsp70*, *Hsp90.1*, *Hsp17.6*, and *Hsp70b* genes compared to ground controls [45]. Therefore, multiple studies demonstrate that various species and cell types may respond to both SMG and space microgravity stress in a conservative way by upregulating Hsp gene expression, but with different signaling output of the HSP response and the effects.

The specific mechanisms underlying how microgravity regulates the expression of Hsp genes remain unclear. However, our data indicate that the expression of Hsp genes increased under simulated microgravity (SMG) conditions, even within a short period of 1–3 h (Figs. 3L and S5D). Previous studies have suggested that factors such as acidic pH, cytoskeleton dynamics, and phase separation may play a role in the regulation of *Hsf1* and *Hsp* gene expression [66–68]. We speculate that these factors may rapidly respond to microgravity, leading to the upregulation of Hsp genes. Investigating the roles of these factors in the regulation of *Hsf1* and *Hsp* genes under microgravity conditions would be an interesting avenue for future research.

The collaboration between HSPs and STAT3 has been documented in various biological processes. A positive feedback loop involving HSPs and STAT3 assumes a pivotal role in sustaining DNA Damage Response (DDR) and preventing DNA damage in Primary Effusion Lymphoma (PEL) cells [69]. The Stat3 inhibitor, Stattic, impedes the acquisition of thermotolerance by suppressing mild heat shock-induced Stat3 phosphorylation and Hsp105 expression [70], thereby implicating the regulatory influence of STAT3 on Hsp105. Knocking down *HSP90* in HEK293 Ob-Rb cell line attenuates leptin-induced STAT3 signaling associated with anorexia [71].

In the present investigation, SMG stress upregulates HSP gene expression, thereby augmenting the activity of key pathways and the expression of core TFs in ESCs (Figs. 3 and S3). Comparative analysis reveals that the upregulation of HSP gene expression occurs within a few hours of SMG treatment (Fig. 3L), while the activation of LIF/STAT3 and its target genes becomes significant after 2–3 days of culture under SMG conditions (Fig. S2A). The investigation into whether the heightened activity of STAT3 collaboratively engages with HSPs to contribute to the self-renewal of ESCs poses an intriguing question warranting further exploration.

Due to technical limitations and the high costs associated with conducting detailed mechanistic studies in true spaceflight-induced microgravity, it is currently challenging to investigate the precise mechanisms involved. Nevertheless, our study has revealed a novel mechanism by which SMG regulates the self-renewal and differentiation of ESCs, as depicted in Fig. S6. Specifically, SMG-induced stress upregulates the expression of Hsp genes, resulting in increased Wnt pathway activity and enhanced expression of core pluripotency factors in both mouse and human ESCs. It is noteworthy that the LIF/STAT3 pathway, which is critical for maintaining mouse ESCs, is not required for the maintenance of human ESCs, yet SMG still increased its activity in mouse ESCs. Furthermore, during ESC differentiation, SMG promotes the differentiation of both human and mouse ESCs into mesoendoderm by activating the Wnt pathway and subsequently upregulating the expression of *Tbx3*.

In addition to microgravity, it is important to consider that mammalian cells cultured under simulated microgravity also experience other physical factors, such as shear stress, oxygen diffusion, and hydrostatic loading [23]. The mechanism proposed in this study is consistent with some preliminary data collected from experiments conducted under true space microgravity conditions. It would be intriguing to verify this mechanism by conducting the experiments described in this study under actual space microgravity in future research.

## Material and Methods

### Cell culture

Mouse E14 ESCs were cultured in DMEM (Hyclone) supplemented with 10% FCS (Gibco), 1xNEAA (Gibco), 1 mM sodium pyruvate (Gibco), 0.1 mM 2-mercaptoethanol (Sigma), 2 mM L-glutamine (Gibco), and LIF (Millipore) on 0.1% gelatin (Sigma) coated plates. 150 µM of HSP inhibitor (sc-221709, Santa Cruz) and 250 nM of 17-AAG (sc-0335, Beyotime) were added to ES medium to inhibit the induction of HSP proteins. Human ESCs were cultured as previously described [72].

A three-dimensional (3D) clinostat (SM-31, Center for Space Science and Applied Research, Chinese Academy of Sciences) was used to simulate microgravity at 37 °C at a random rotating speed of 0–10 r.p.m. [73].

### ESC differentiation by embryoid body (EB) induction

ESCs dissociated with 0.05% trypsin were centrifuged and re-suspended in ES medium without LIF. About 6–8 × 10^5^ of ESCs were then plated in a 25 ml cell culture flask and incubated at 37 °C with 5% CO_2_ to induce the formation of EBs in about 43 ml of ES medium without LIF or until the flask is fully filled. EBs at indicated days were collected for qPCR analysis.

### Apoptosis assay

A total of 1 × 10^6^ cells were washed with PBS then labeled with 7-AAD (BD Pharmingen) and Annexin-V (Biolegend) for 15 min. Cells were washed with PBS and measured using a FACS Canto II flow cytometer (BD Biosciences, San Jose, CA). Analysis was performed using Flowjo software (TreeStar, Ashland, OR).

### Metabolic assay

Cellular oxygen consumption rate (OCR) of mESCs under SMG and corresponding 1 g control were detected by Seahorse XFe24 extracellular flux analyzer with Cell Mito Stress Test Kit (cat# 103010-100) following manufacturer’s protocols. Basal respiration (BR), maximal respiration (MR), proton leak (PL) and ATP production (AP) were calculated from oxygen consumption rate (OCR) values using Wave Desktop software.

Specifically, detection plates were hydrated with 800 µl/well of sterile water in a 37 °C, CO_2_-free incubator overnight, and then coated with poly-lysine overnight at 4 °C. The next day, mESCs cultured for 4 days under simulated microgravity and corresponding 1 g controls were washed twice with OCR, counted, and resuspended to 3 × 10^5^ cells/100 µl. The 100 µl of cell suspension was added to one-well of a 24-well assay plate (seeded 3 × 10^5^ cells/well). The plates were centrifuged at 200 g for 2 min, and incubated in a non-CO_2_ incubator at 37 °C for 30 min. Then 400 µl of ES medium was added slowly per well for one hour’s culture.

Final concentrations of reagents for OCR test: oligomycin 1 µM, FCCP 1 µM, Antimycin A/Rotenone 0.5 µM.

### Western blot analysis

Protein samples were fractionated on 10% SDS-PAGE gels, electroblotted onto PVDF membranes (Millipore), and membranes probed sequentially with respective antibodies. Blots were incubated with secondary antibodies and developed with ECL Plus (Bio-Rad). The antibody information was provided in supplementary Table S1. The full-length, uncropped original Western blots have been included in Table S2.

### Quantitative RT-PCR

Total RNA was isolated with FastPure Cell/Tissue Total RNA Isolation Kit V2 (Vazyme). cDNA was synthesized with HiScript II Q RT SuperMix (Vazyme). Real-time PCR was performed with Taq Pro Universal SYBR qPCR Master Mix (Vazyme). Gene expression was determined relative to Gapdh transcript levels. Standard deviation was calculated from PCR triplicates. Error bars give the SD of three technical qPCR replicates from a representative experiment.

### Generation of Tbx3, Hsf1 and Hsph1 knockout ESC clones

2 µg of gRNA and 2 µg of Cas9 plasmids were electroporated to ESCs. After 7 days’ selection with 175 µg/ml of G418, colonies were picked up for genotyping and confirmed by Sanger sequencing and Western blot analysis.

### RNA-seq, ChIP-seq and ChIP-qPCR experiments

RNA-seq, ChIP-seq and ChIP-qPCR experiments were performed as previously described [48, 74]. Mouse and human ESCs were cultured at 1 g and SMG conditions for 4 days for RNA-seq and indicated time for ChIP experiments. For ChIP-qPCR experiments, bound regions were detected by using paired primers given in Table S1.

### Data analysis

Reads were trimmed by fastp [75] to remove adaptors at the 3’ end. The trimmed reads were aligned against the mouse genome (build mm10) using hisat2 [76]. Duplicates were removed using the Picard tools (https://broadinstitute.github.io/picard/). For ChIP-seq, peak calling was performed with MACS2 [77] using the “–nomodel –extsize 200” flags. For ATAC-seq, peak calling was performed with MACS2 using the “–nomodel –shift -100 –extsize 200” flags. For RNA-seq, gene expression was quantified and differentially expression was performed using cuffdiff [78]. To find differentially accessible peaks from the ATAC-seq data, a union set of peaks were created by merge the peaks from either the normal or microgravity conditions. Then reads from each replicate of each condition were counted against the union peaks using coverageBed from the bedtools suite [79]. The count table was used as an input for DESeq2 [80] to identify statistically significant differential peaks. To define chromatin states using histone modification ChIP-seq data from each condition, ChromHMM [81] was used with default parameters.

### GO analysis

GO analysis for enriched biological processes was performed using Metascape (http://metascape.org) to find significantly enriched terms (*P* value % 0.01).

### Supplementary information

Supplemental files

Table S1

Table S2

Table S3

Table S4

## Data Availability

The raw sequencing data have been deposited at ArrayExpress under the accession number E-MTAB-12389 (RNA-seq) and E-MTAB-12390 (ChIP-seq).
