# Supplementary material for: Conserved mechanisms of self-renewal and pluripotency in mouse and human ESCs regulated by simulated microgravity using a 3D clinostat
Source: Cell Death Discov. 2024 Feb 9;10:68. doi: 10.1038/s41420-024-01846-2 (PMC10858198; doi:10.1038/s41420-024-01846-2)
Supplement: Supplementary file 1 — Supplemental files [file 41420_2024_1846_MOESM1_ESM.docx]

**Supplemental Information**

**Conserved Mechanisms of Self-Renewal and Pluripotency in Mouse and Human ESCs Regulated by Simulated Microgravity Using a 3D Clinostat**

Ying Ye, Wenyan Xie, Zhaoru Ma, Xuepeng Wang, Yi Wen, Xuemei Li, Hongqian Qi, Hao Wu, Feng Li, Jinnan An, Yan Jiang, Xinyi Lu, Shijun Hu, Elizabeth A. Blaber, Xi Chen, Lei Chang, Wensheng Zhang

**SUPPLEMENTAL FIGURES**


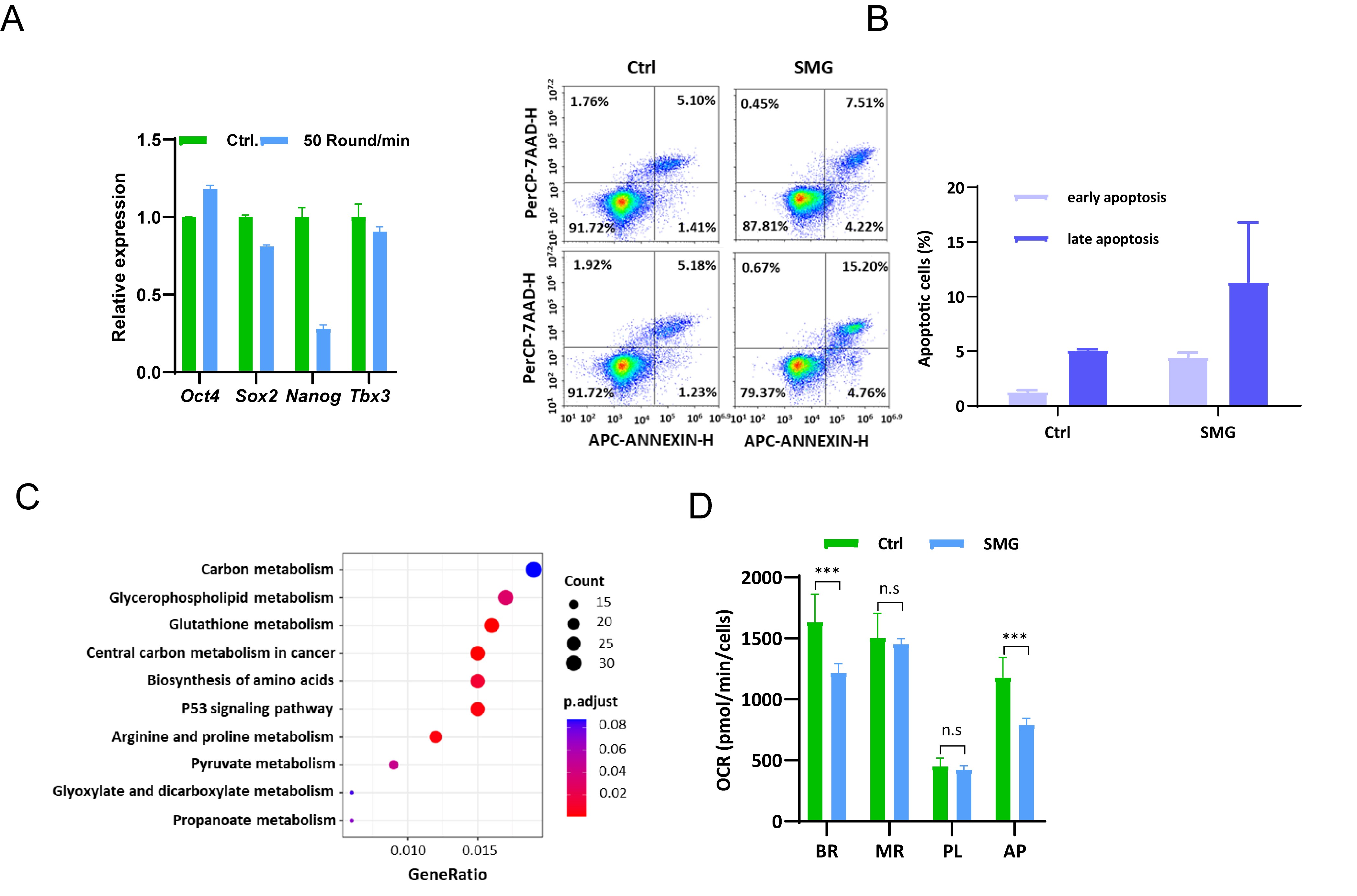


**Fig. S1 (Related to Fig. 1) Simulated microgravity induces apoptosis and metabolic alterations. A** Transcript levels of pluripotency-associated genes in mESCs cultured for 4 days in mESCs cultured within flasks placed on a horizontal shaker operating at 50 rounds per minute were determined through qPCR analysis. (n=3 independent experiments, **p < 0.01,***p < 0.001, n.s, not significant). **B** Representative fluorescence-activated cell sorting (FACS) plots of Annexin V and 7-aminoactinomycin D (7-AAD) levels in mESCs cultured at 1g and SMG conditions for 4 days. The percentages of cells exhibiting distinct levels of apoptosis markers are denoted in brackets (n=3 independent experiments). **C** Metabolic pathways exhibiting significant enrichment: Bubble plot showcasing KEGG pathway enrichment analysis of Differentially Expressed Genes (DEGs) in mESCs cultured under 1g and SMG conditions. The "Count" column represents the number of DEGs enriched in each pathway. "GeneRatio" indicates the ratio of enriched DEGs to background genes, while "p.adj" represents the p-value adjusted using the 'BH' method. **D** Comparison of Oxygen Consumption Rate (OCR) in mESCs: Mito stress test measurement of OCR in mESCs cultured under 1g and microgravity conditions. The following parameters were calculated from the Mito stress test results in **Fig. 1F**: Basal Respiration (BR), Maximal Respiration (MR), Proton Leak (PL), and ATP Production (AP). The data represents 3 independent experiments (n=3), with statistical significance indicated as ***p < 0.001, and non-significant results denoted as n.s.

**
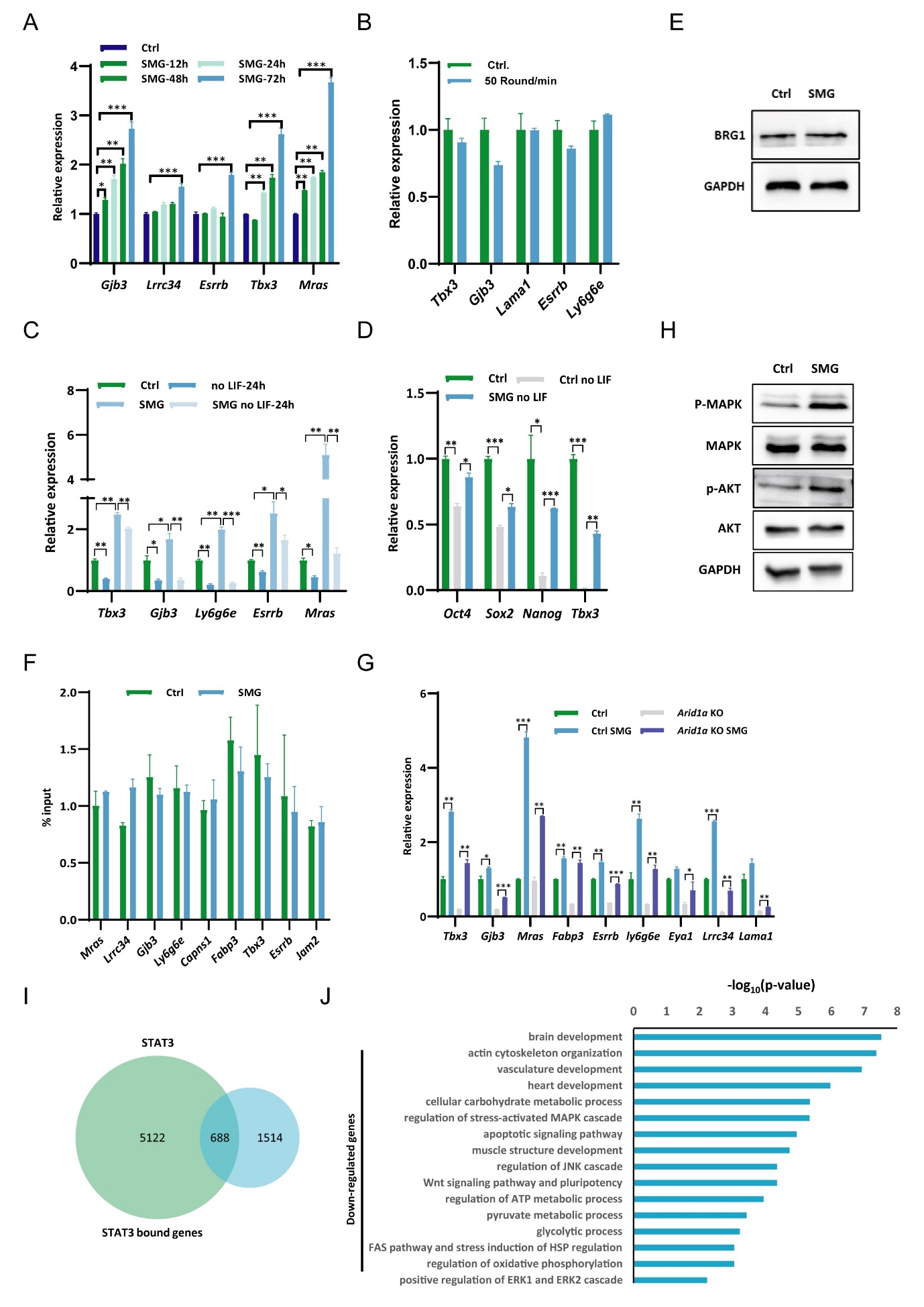
**

**Fig. S2 (Related to Fig. 2): Elevated activity of the LIF/STAT3 pathway in mESCs under SMG condition. A** qPCR analysis of transcript levels of LIF/STAT3 target genes *Gjb3*, *Lrrc34*, *Esrrb*, *Tbx3*, and *Mras* in mESCs cultured for 12h, 24h, 48 and 72h under microgravity (SMG) and normal gravity (1g) conditions. The data represents 3 independent experiments (n=3), with statistical significance indicated as *p < 0.05, **p < 0.01, and ***p < 0.001. **B** qPCR analysis of transcript levels of LIF/STAT3 target genes Tbx3, Gjb3, Lrrc34, Esrrb, Tbx3, and Mras in mESCs cultured within flasks placed on a horizontal shaker operating at 50 rounds per minute were determined through qPCR analysis. The data represents 3 independent experiments (n=3), with statistical significance indicated as *p < 0.05, **p < 0.01, and ***p < 0.001. **C** qPCR analysis of transcript levels of LIF/STAT3 target genes *Tbx3*, *Gjb3*, Lama1, Esrrb, and *Ly6g6e* in mESCs cultured for 4 days under microgravity (SMG) and normal gravity (1g) conditions, with and without LIF supplementation. The data represents 3 independent experiments (n=3), with statistical significance indicated as *p < 0.05, **p < 0.01, and ***p < 0.001. **D** qPCR analysis of transcript levels of *Oct4*, *Sox2*, *Nanog*, and *Tbx3* in mESCs cultured for 4 days in ES medium with and without LIF, under normal gravity (1g, Ctrl) and microgravity (SMG) conditions. The data represents 3 independent experiments (n=3), with statistical significance indicated as *p < 0.05, **p < 0.01, and ***p < 0.001. **E** Western blot analysis of BRG1 levels in mESCs cultured for 4 days under normal gravity (1g) and microgravity (SMG) conditions. GAPDH was used as a loading control. The data represents 3 independent experiments (n=3). **F** ChIP-qPCR analysis of LIF/STAT3 target genes *Mras*, *Lrrc34*, *Gjb3*, *Ly6g6e*, *Capns1*, *Fabp3*, *Tbx3*, *Esrrb*, and *Jam2* in mESCs cultured under normal gravity (1g) and microgravity (SMG) conditions, performed using BRG1 antibody. The data represents 3 independent experiments (n=3). **G** qPCR analysis of transcript levels of LIF/STAT3 target genes *Tbx3*, *Gjb3*, *Mras*, *Fabp3*, *Esrrb*, *Ly6g6e*, *Eya1*, *Lrrc34*, and *Lama1* in wild-type (WT) and Arid1a knockout (KO) mESCs cultured for 4 days under microgravity (SMG) and normal gravity (1g) conditions. The data represents 3 independent experiments (n=3), with statistical significance indicated as *p < 0.05, **p < 0.01, and ***p < 0.001. **H** Western blot analysis of MAPK and p-MAPK, AKT and p-AKT levels in mESCs cultured for 4 days under normal gravity (1g) and microgravity (SMG) conditions. GAPDH was used as a loading control. The data represents 3 independent experiments (n=3). **I** Venn diagram illustrating the overlapping genes that are down-regulated in mESCs cultured for 4 days under microgravity (SMG) conditions and bound by STAT3 protein. The diagram showcases the shared number of genes between the two conditions. **J** GO ontology analysis revealing biological processes associated with down-regulated STAT3 target genes under microgravity (SMG) conditions. The analysis identifies the functional categories enriched among the down-regulated genes.

**
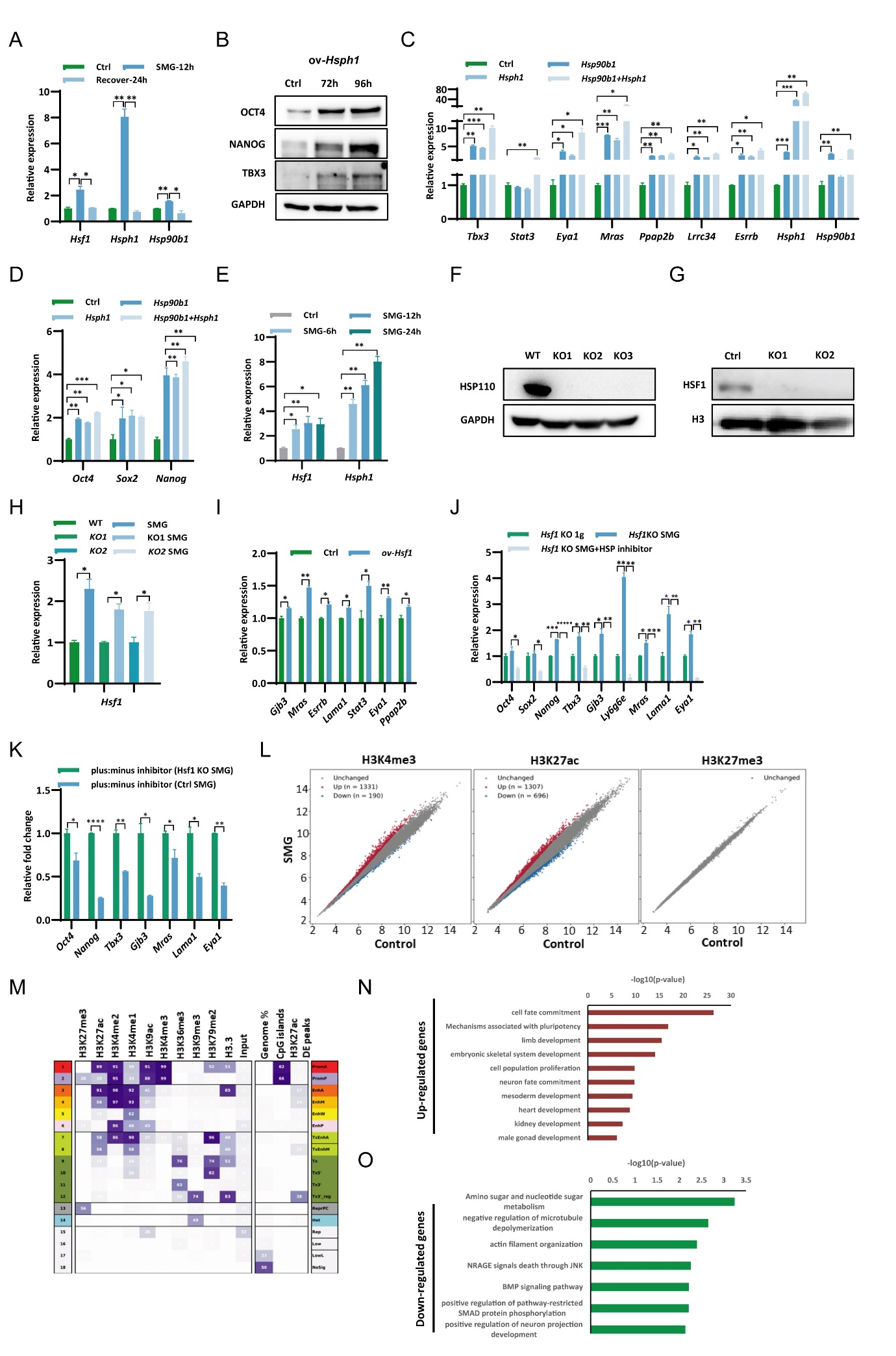
**

**Figure S3 (Related to Fig.3): Regulation of LIF/STAT3 target genes and pluripotency genes by HSF1/HSP proteins. A** Transcript levels of Hsf1, Hsph1, and Hsp90b1 were assessed using qPCR analysis in mESCs. The cells were exposed to three distinct culture conditions: SMG, normal gravity (1g), and a 24-hour culture at 1g following a 12-hour culture under SMG. (n=3 independent experiments, *p < 0.05,**p < 0.01). **B** Western blot analysis of OCT4, NANOG, and TBX3 levels in mESCs overexpressing *Hsph1* for 72 and 96 hours, with GAPDH as the loading control. (n=3 independent experiments). **C** qPCR analysis of transcript levels of LIF/STAT3 target genes *Tbx3*, *Stat3*, *Eya1*, *Mras*, *Ppap2b*, *Lrrc34*, *Esrrb* in mESCs overexpressing *Hsp90b1*, *Hsph1*, and *Hsp90b1*/*Hsph1* for 72 hours. (n=3 independent experiments, *p < 0.05,*p<0.01, ***p < 0.001).

**D** qPCR analysis of transcript levels of *Oct4*, *Sox2* and *Nanog* in mESCs overexpressing *Hsp90b1*, *Hsph1* and *Hsp90b1/Hsph1* for 72 hours. (n=3 independent experiments, *p < 0.05,*p<0.01, ***p < 0.001). **E** qPCR analysis of transcript levels of *Hsf1* and *Hsph1* in mESCs cultured under SMG for 0, 6, 12, and 24 hours. (n=3 independent experiments, *p < 0.05,**p<0.01). **F** Western blot analysis of HSP110 levels in WT and *Hsph1* mESCs, with GAPDH as the loading control. (n=3 independent experiments). **G** Western blot analysis of HSF1 levels in WT and *Hsf1* KO mESCs, with GAPDH as the loading control. (n=3 independent experiments). **H** qPCR analysis of transcript levels of *Hsf1* in WT and *Hsph1* KO mESCs cultured under 1g and SMG conditions for 1 day. (n=3 independent experiments, *p < 0.05). **I** qPCR analysis of transcript levels of LIF/STAT3 target genes *Gjb3*, *Mras*, *Esrrb*, *Lama1*, *Stat3*, *Eya1*, and *Ppap2b* in WT mESCs and mESCs overexpressing the *Hsf1* gene for a duration of 3 days. (n=3 independent experiments, *p < 0.05, **p<0.01).

**J** qPCR analysis of transcript levels of indicated genes in Hsf1 KO mESCs cultured under 1g and SMG conditions for 3.5 days, followed by treatment with HSP inhibitors for an additional 10 hours. (n=3 independent experiments, *p < 0.05, *p<0.01, ***p < 0.001, ****p<0.0001). **K** Relative fold change of indicated gene expression resulting from HSP protein inhibition in WT and *Hsf1* KO mESCs cultured under SMG condition. WT and *Hsf1* KO mESCs were cultured under SMG condition with and without HSP inhibitors. The fold change in expression of the indicated genes resulting from HSP protein inhibition was calculated separately for WT mESCs (**Fig. 3G**) and Hsf1 KO mESCs (**Fig. 3J**). A comparison of the relative fold change in gene expression between WT and Hsf1 KO mESCs is shown in Fig. S3K. (n=3 independent experiments, *p < 0.05, *p<0.01, ***p < 0.001). **L** Scatter plot displaying the log RPKM values of H3K4me3 (left), H3K27ac (middle), and H3K27me3 (right) in mouse embryonic stem cells (mESCs). Significant differential peaks identified by DESeq2 (|log2FC| > 1 and q value < 0.01) are highlighted in red (upregulated) and blue (downregulated) colors. **M** Chromatin states defined by ChromHMM using the ChIP-seq data of the H3K27ac. Heatmap representation of the emission probability of each histone modifications (left) and the enrichment of different genomic segments (right). **N**, **O** Enriched GO terms identified for genes associated with increased (**N**) and decreased (**O**) H3K4me3 in mESCs cultured for 11 hours under SMG and 1G (normal gravity) environments.


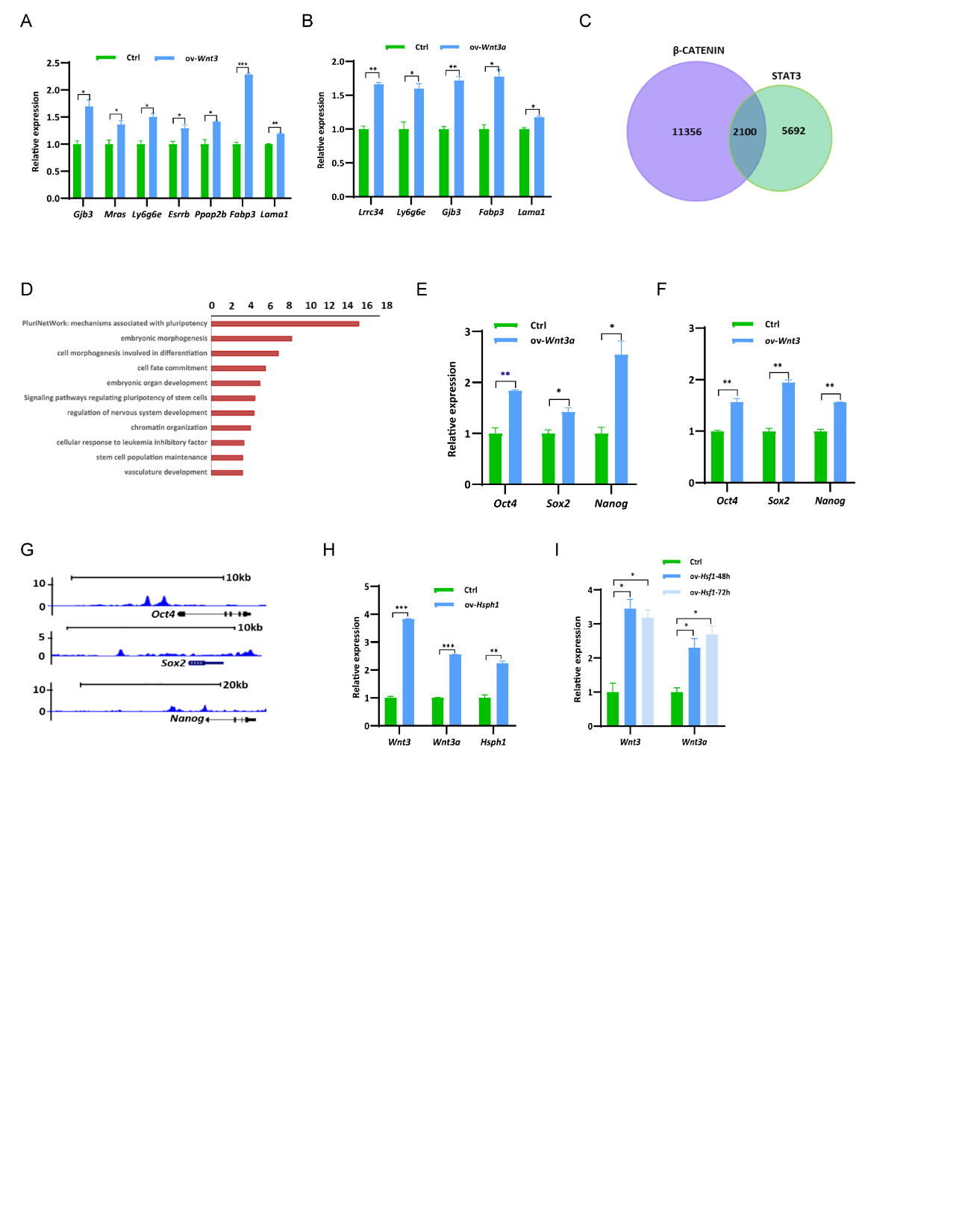


**Fig. S4 (Related to Fig. 4): The activation of the Wnt signaling pathway under SMG conditions upregulates the expression of both LIF/STAT3 target genes and pluripotency factors. A**, **B** qPCR analysis of Tbx3 expression in WT and mESCs with overexpression of Wnt3 (**A**) or Wnt3a (**B**) for 3 days. (n=3 independent experiments, *p < 0.05,**p<0.01, ***p < 0.001). **C** The Venn diagram illustrates the overlap in the number of peaks bound by β-CATENIN and STAT3, as determined by β-CATENIN ChIP-seq and STAT3 ChIP-seq analyses. **D** GO analysis of genes bound by both STAT3 and β-CATENIN. **E**, **F** qPCR analysis of transcript levels of *Oct4*, *Sox2* and *Nanog* genes in control and mESCs overexpressing *Wnt3a* (**E**) and *Wnt3* (**F**) for 3 days. (n=3 independent experiments, *p < 0.05,**p<0.01). **G** ChIP-seq signal tracks of β-CATENIN at the Oct4, Sox2, and Nanog loci in the genome browser. **H**, **I** qPCR analysis of transcript levels of *Wnt3* and *Wnt3a* genes in both control and mESCs overexpressing *Hsph1* for 3 days (**H**) as well as Hsf1 for 2 days and 3 days (**I**), respectively. (n=3 independent experiments, *p < 0.05, **p< 0.01, ***p < 0.001).

**
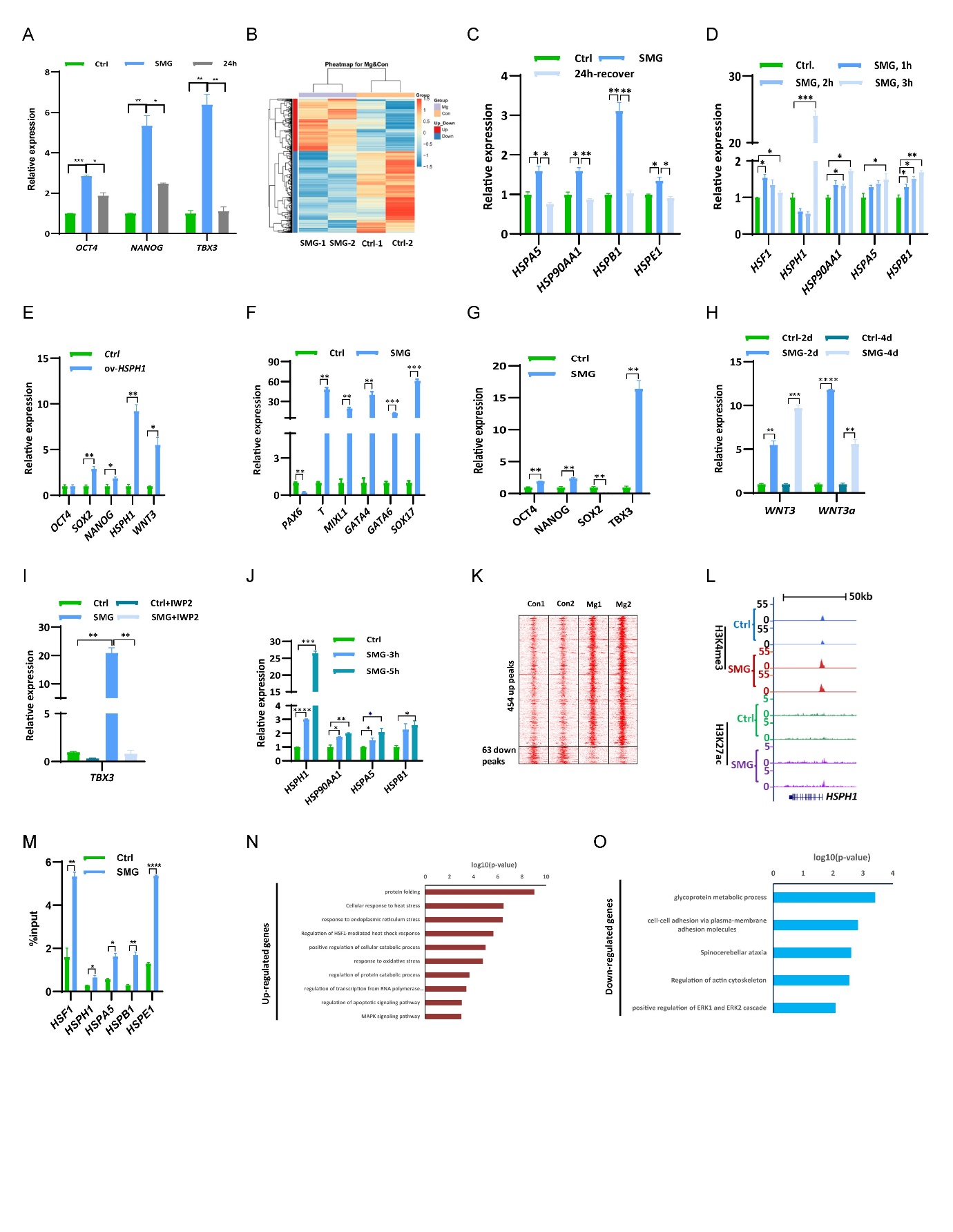
**

**Fig. S5 (Related to Fig. 5): SMG affects the self-renewal and differentiation of hESCs. A** qPCR analysis of transcript levels of pluripotency genes *OCT4* and *SOX2* in hESCs cultured under SMG, 1g condition, 24 hours’ culture at 1g condition after cultured under SMG condition. **B** Heat map depicts the dysregulated expression in hESCs cultured under 1g and SMG conditions. **C** qPCR analysis of transcript levels of *HSPA5*, *HSP90AA1*, *HSPB1* and *HSPE1* in hESCs cultured under SMG and normal gravity (1g) conditions for a duration of 4 days, as well as 24 hours of culture at 1g following 3 days of SMG. **D** qPCR analysis of transcript levels of *HSH1*, *HSPH1*, *HSP90AA1*, *HSPA5* and *HSPB1* in mESCs cultured under normal gravity (1g) and simulated microgravity (SMG) conditions for 1, 2 and 3 hours. (n=3 independent experiments, *p < 0.05). **E** qPCR analysis of transcript levels of *OCT4*, *SOX2*, *NANOG* and *WNT3* genes in both control and hESCs overexpressing the *HSPH1* gene. **F** qPCR analysis of transcript levels of endoderm marks *GATA4*, *GATA6* and *SOX17*, mesoderm markers *T* and *MIXL1*, neuronal ectoderm marker *PAX6* in day 4 EBs induced under 1g and SMG conditions. **G** qPCR analysis of transcript levels of pluripotency genes *OCT4*, *SOX2*, *NANOG* and *TBX3* in day 4 EBs induced under 1g and SMG conditions. **H** qPCR analysis of transcript levels of *WNT3* and *WNT3a* in day 2 and day 4 EBs induced from hESCs under 1g and SMG conditions. **I** qPCR analysis of transcript level of *TBX3* in day 2 EBs induced under 1g and SMG conditions, with and without the presence of 10μg/ml of IWP2. **J** qPCR analysis of transcript levels of *HSPH1*, *HSP90AA1*, *HSPA5* and *HSPB1* genes in hESCs cultured under normal gravity (1g) condition, as well as after 3 and 5 hours of culture under SMG condition. **K** The heatmap depicts the altered H3K27ac signals in hESCs cultured under both normal gravity and SMG conditions for a duration of 5 hours. **L** Genome browser view of H3K4me3 and H3K27ac at the *HSPH1* locus in hESCs. **M** ChIP-qPCR analyses of the HSF1, HSPH1, HSPA5, HSPB1, and HAPE1 loci from hESCs cultured under 1g and SMG conditions for 2 days were carried out with H3K4me3 antibody. **N**, **O** The enriched gene ontology (GO) terms associated with increased H3K4me3 (**N**) and decreased H3K27ac (**O**) in hESCs cultured under SMG and 1G conditions, respectively.


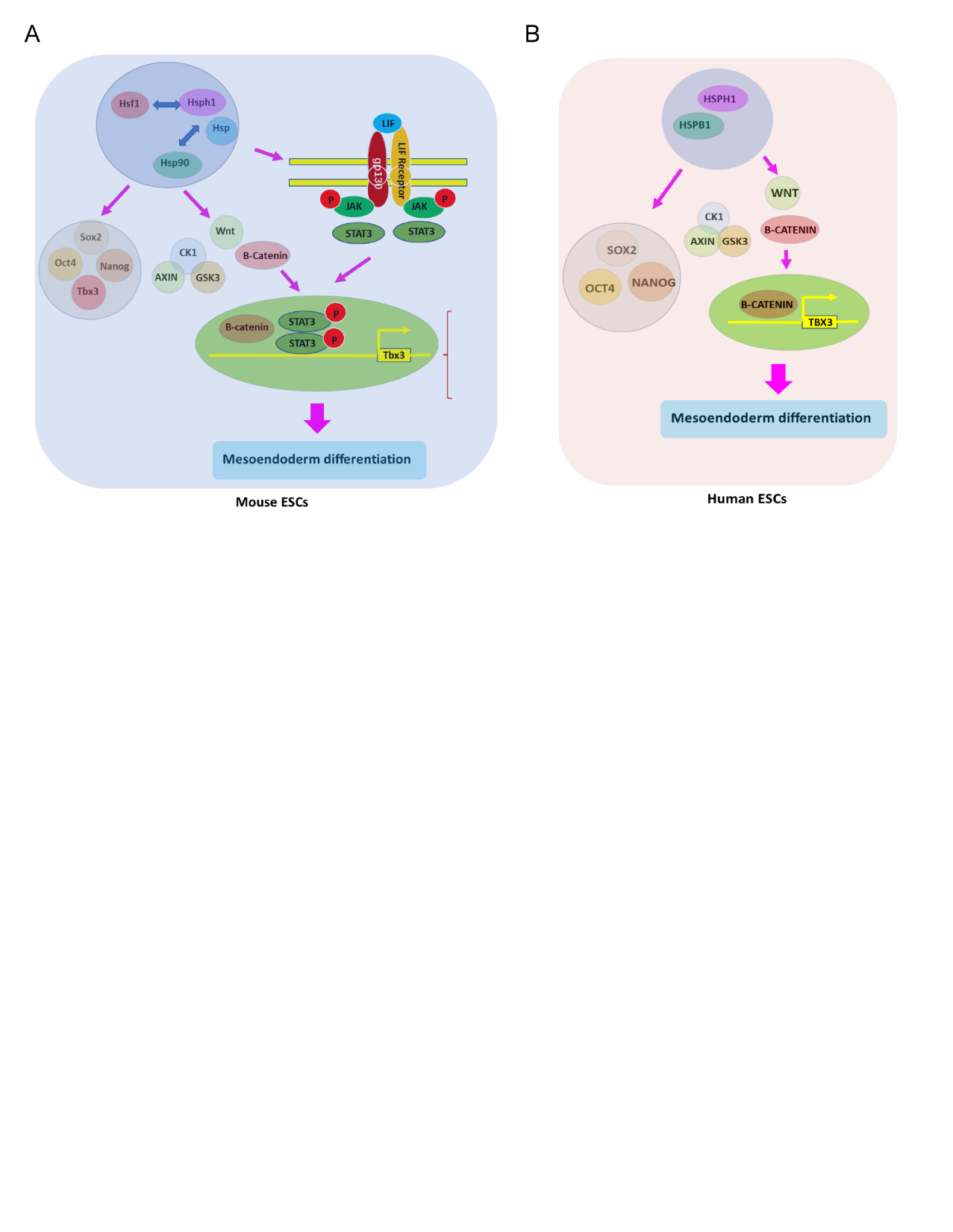


**Fig. S6.** **Schematic summary of findings showing the effects of SMG on the self-renewal and differentiation of both mouse and human ESCs. A** Simulated microgravity (SMG) induces an upregulation of *Hsf1* and *Hsp* genes, resulting in an increased activation of LIF/STAT3 and Wnt signaling pathways in mESCs. This activation, in turn, leads to enhanced expression of core pluripotency genes and LIF/STAT3 target genes in mESCs. Notably, the collaborative action of Wnt and STAT3 signaling pathways regulates the expression of *Tbx3* gene, thereby promoting the mesoendoderm differentiation of mESCs. **B** Likewise, exposure to SMG resulted in an upregulation of *HSP* genes in hESCs, potentially contributing to the enhanced activation of the Wnt signaling pathway and the expression of pluripotency-associated genes. SMG was found to facilitate the differentiation of hESCs into the mesoendoderm lineage by activating the Wnt signaling pathway and inducing an upregulation of *TBX3* expression.

**Supplementary table legends**

**Table S1.** Primer sequences and antibody Information utilized in the study.

**Table S2.** The full-length, uncropped original Western blots.

**Table S3.** Genes differentially expressed in mouse ESCs cultured under 1g and SMG conditions.

**Table S4.** Genes differentially expressed in human ESCs cultured under 1g and SMG conditions.
