# Supplementary material for: Conserved mechanisms of self-renewal and pluripotency in mouse and human ESCs regulated by simulated microgravity using a 3D clinostat
Source: Cell Death Discov. 2024 Feb 9;10:68. doi: 10.1038/s41420-024-01846-2 (PMC10858198; doi:10.1038/s41420-024-01846-2)
Supplement: Supplementary file 2 — Table S1 [file 41420_2024_1846_MOESM2_ESM.docx]

| **qPCR Primers For mouse genes** | | |
| --- | --- | --- |
| **Gene（ID）** | **Forward（5’-3’）** | **Reverse（5’-3’）** |
| *Gapdh* | AGAACATCATCCCTGCATCC | CACATTGGGGGTAGGAACAC |
| *Oct4* | GTGGAGGAAGCCGACAACAATGA | CAAGCTGATTGGCGATGTGAG |
| *Sox2* | CAGGAGAACCCCAAGATGCACAA | AATCCGGGTGCTCCTTCATGTG |
| *Nanog* | TGGTCCCCACAGTTTGCCTAGTTC | CAGGTCTTCAGAGGAAGGGCGA |
| *Tbx3* | CTACGGGGGAGCAATGGATG | AGTTTAGTATAGTAAATCCG |
| *Klf4* | GTGCAGCTTGCAGCAGTAAC | AGCGAGTTGGAAAGGATAAAGTC |
| *Klf2* | CCAAGAGCTCGCACCTAAAG | GTGGCACTGAAAGGGTCTGT |
| *Klf5* | TTTCCCCCGTCACCACCAA | CTTCTGTTGTATCTGACAGG |
| *Stat3* | CTACGGGGGAGCAATGGATG | AGTTTAGTATAGTAAATCCG |
| *Lama1* | GATCCCATTTATGTGGGTGG | CTTGTAGGTCAAAGGCTCGG |
| *Gjb3* | GAATCAAGGCCAGGTCTGAG | ATGCCGTGGAGTACTGGTTC |
| *Ly6g6e* | TGTTACACCTGCAGCTTTGC | TGCATAGGTCCTGCTCACAG |
| *Mras* | AGAGACCAGTGCCAAGGACC | TCAGTGCAGTTAGGCAGTGG |
| *Fabp3* | GCAAACTCATCCATGTGCAG | TCAGAGGGGAAAACCATGAG |
| *Esrrb* | GGTGCGCAGGTACAAGAAAC | AGGAAGAGTTTGTGCATGGG |
| *Ppap2b* | GCCTTCTACACGGGATTGTC | TGTTCCTGTCGATGATGTCC |
| *Lrrc34* | TTGCACTTTCCCAGTCAATG | TTTCTCCATAAGTCGGTGCC |
| *Capns1* | CCGGACAGATCCAAGTGAAC | TGGCTCCATAGTGAGCTGTG |
| *Eya1* | GTGTGGAAGAAGAGCAAGGG | CAAGCCTGCTGGGTTATCTC |
| *Hspa1b* | CATCAGTGGGCTGTACCAGG | GCCGCCGAAAGGAGCC |
| *Hsp90b1* | CCGAGAAGGCTCAAGGACAG | TCACTTCAGCTTGGAAGGCG |
| *Hsph1* | GTTGGGCTAGACGTAGGCTC | GCTGGTTTTTGGCTGCAACT |
| *Hsf1* | GTTCCAGCATCCTTGTTTCTTG | GACACTGTCCTGGCGTATTT |
| *T* | GAGTCAAGACTCCTGGAAGGTG | CCACTCGCAGTTCGCGTTC |
| *Mixl1* | ATCCGCCCGGACCCTCCAAA | TCGGTTCTGGAACCACACCTGGA |
| *Sox17* | CGCACGGAATTCGAACAGTA | GTCAAATGTCGGGGTAGTTG |
| *Gata6* | TTGCTCCGGTAACAGCAGTG | GTGGTCGCTTGTGTAGAAGGA |
| *Wnt3* | TGGGCCTGTCTTGGACAAA | GCGATGGCATGCACGAA |
| *Wnt3a* | CATGAACCGTCACAACAATGAG | CACTTCACAGCTGCCAGATA |

| **qPCR Primers For human genes** | | |
| --- | --- | --- |
| **Gene（ID）** | **Forward（5’-3’）** | **Reverse（5’-3’）** |
| *GAPDH* | GTGGACCTGACCTGCCGTCT | GGAGGAGTGGGTGTCGCTGT |
| *OCT4* | AGCGAACCAGTATCGAGAACC | CTGATCTGCTGCAGTGTGGGT |
| *SOX2* | AGTGTTTGCAAAAGGGGGAAAGTAG | CCGCCGCCGATGATTGTTATTATT |
| *NANOG* | CAGAAGGCCTCAGCACCTAC | ATTGTTCCAGGTCTGGTTGC |
| *TBX3* | GGACACTGGAAATGGCCGAAGA | GCTGCTTGTTCACTGGAGGACT |
| *GATA6* | TTCGTTTCCTGGTTTGAATTCC | TGCAATGCTTGTGGACTCTAC |
| *GATA4* | GTGTCCCAGACGTTCTCAGTC | GGGAGACGCATAGCCTTGT |
| *SOX17* | CGCACGGAATTTGAACAGTA | GGATCAGGGACCTGTCACA |
| *T* | ACCCAGTTCATAGCGGTGAC | CCATTGGGAGTACCCAGGTT |
| *MIXL1* | AGCTGCTGGAGCTCGTCTTC | TGGAAGGATTTCCCACTCTG |
| *PAX6* | CACCTACAGCGCTCTGCCGC | CCCGAGGTGCCCATTGGCTG |
| *HSF1* | GGAAAGTGGTCCACATCGAG | TTCACTCTCCCGCAGGATGG |
| *HSP110* | AGGAGTTCCATATCCAGAA | CAGCTCAACATTCACCAC |
| *HSPA5* | CACAGTGGTGCCTACCAAGA | TGTCTTTTGTCAGGGGTCTTT |
| *HSP90AA1* | CTTGACCAATGACTGGGAAGAT | CACGTCGTGGGACAAATAGAA |
| *HSPB1* | CTGACGGTCAAGACCAAGGATG | GTGTATTTCCGCGTGAAGCACC |
| *HSPE1* | TGGATCGGGTTCTAAAGGAAAG | CTACTTTGGTGCCTCCATATTCT |
| *WNT3* | GGAGAAGCGGAAGGAAAAATG | GCACGTCGTAGATGCGAATACA |
| *WNT3A* | CCTGCACTCCATCCAGCTACA | GACCTCTCTTCCTACCTTTCCCTTA |

| **ChIP-qPCR Primers For mouse genes** | | |
| --- | --- | --- |
| **Gene（ID）** | **Forward（5’-3’）** | **Reverse（5’-3’）** |
| *Tbx3-DE* | CTCAATTCTCTGCCCTTTGG | AAGTCCGGGAAACTCCAATC |
| *Gjb3* | TGAAGGACGATAGCACAAGG | TGGCCACTCCTAGAACATCA |
| *Ly6g6e* | CTAGAACCGGGAACAGCTTG | CTTGCCTTGAGGGTCTTCTG |
| *Mras* | GACGCCTTAAAGCGACCG | ACAGACTGCAGATCCGTGTG |
| *Esrrb* | CAGCCTTCCTACTCCACAGG | AGTGACAGAATCGAGCTGGG |
| *Lrrc34* | CTCTCCTCACGAAGACAGGG | GGAGCGACTCTGGTTACTGG |
| *Jam2* | GAGGAGAGACATGGCGAGAC | AAGGGTGGAAGAGGGTTGAG |
| *Capns1* | ACTGGAGTGTCCCGTCTCAC | TGCAATGGAGGCTCTAGGAC |
| *Fabp3* | CGGTACCTGGAAGCTAGTGG | CTGGACTGGCTAGGAGCTTG |
| *Esrrb* | CAGCCTTCCTACTCCACAGG | AGTGACAGAATCGAGCTGGG |
| *Tbx3*  *(for IE)* | CAGGGTTACATAGACAATAAACCG | ACAGCCTGACTACAGAGTGAAAGT |
| *Tbx3-p-1*  *(for Promoter)* | GACATAAACGCAGGACAGG | CATAGGCGTGGTTTTCACAG |
| *Tbx3-p-2*  *(for Promoter)* | TCTTTCTCTCCCTCCCTCTAC | AGGATCAAGAAGACGGGTG |

| **ChIP-qPCR Primers For human genes** | | |
| --- | --- | --- |
| **Gene（ID）** | **Forward（5’-3’）** | **Reverse（5’-3’）** |
| *hHSPH1*  *(for Promoter)* | TTAAGCCGACGCTGTTCTAC | ACATCGCTTCTGAGGGAAAG |
| *hHSF1*  *(for Promoter)* | CTCCGCCTATTCCCTCCTT | ACGAGGGTCCACAGCTT |
| *hHSPB1*  *(for Promoter)* | GCCCTCAAACGGGTCATT | GTCATGCTGGCTGACTCTG |
| *hHSP90AA1*  *(for Promoter)* | CAAAGAATCCAGCCGCAAG | CGGTGGGTCTGGAGAATC |
| *HSPA5*  *(for Promoter)* | AGGCATTTCCGCTGGTAAC | GAAGGGAGAACAAGCAGTAGAG |
| *HSPE1*  *(for Promoter)* | CTTGACCCAGCGTTTCCT | CCCGATTCCTTACGTCTGTTAG |

| **sgRNA sequences** | |
| --- | --- |
| **Gene (ID)** | **gRNA sequence** |
| *Hsf1* | acaccatcatagtttcactg(sgRNA1) |
| *Hsf1* | agtgaagcaagcatagcatc(sgRNA2) |
| *Hsf1* | ACTGGCCCTGGTCAAACACG(sgRNA3) |
| *Hsf1* | TGTTGTTGTGCTTGAAGTAC(sgRNA4) |
| *Hsp110* | TTTAGGTCAGTCATATCATT(sgRNA1) |
| *Hsp110* | AAAACCAGGTACGTTCATCT(sgRNA2) |
| *Hsp110* | CACACATGCGGCCGTTTCT(sgRNA3) |
| *Hsp110* | GGTACGTTCATCTGTCTGTC(sgRNA4) |

| **Antibody** | **Source** | **Cat. No.** |
| --- | --- | --- |
| GAPDH | FD | FD0063 |
| H3 | ABClonal | A2348 |
| OCT4 | Santa Cruz | SC5297 |
| SOX2 | Abcam | ab97959 |
| NANOG | Abcam | ab80892 |
| NANOG | CST | 8822 |
| TBX3 | Invitrogen | 42-4800 |
| STAT3 | Santa Cruz | SC8019 |
| p-STAT3 | CST | 9131s |
| p-STAT3 | Santa Cruz | SC8059 |
| GP130 | Santa Cruz | SC655 |
| p-GP130 | Santa Cruz | SC377572 |
| HSP110 | Abclone | A4687 |
| JAK1 | proteintech | 66460-1-IG |
| p-JAK1 | CST | 3331s |
| HSF1 | CST | 4356 |
| p-HSF1 | Abcam | ab115702 |
| β-catenin | CST | D10A8 |
| BRG1 | Santa Cruz | SC17796 |
| MAPK | CST | 4696s |
| p-MAPK | CST | 9101s |
| AKT | CST | 4691s |
| p-AKT | Affbiotech | AF0016 |
| H3K4me3 | Abcam | ab8580 |
| H3K27me3 | Abcam | ab6002 |
| H3K27ac | Abcam | ab177178 |
