# Supplementary material for: Conserved mechanisms of self-renewal and pluripotency in mouse and human ESCs regulated by simulated microgravity using a 3D clinostat
Source: Cell Death Discov. 2024 Feb 9;10:68. doi: 10.1038/s41420-024-01846-2 (PMC10858198; doi:10.1038/s41420-024-01846-2)
Supplement: Supplementary file 3 — Table S2 [file 41420_2024_1846_MOESM3_ESM.pdf]

Figure 1C

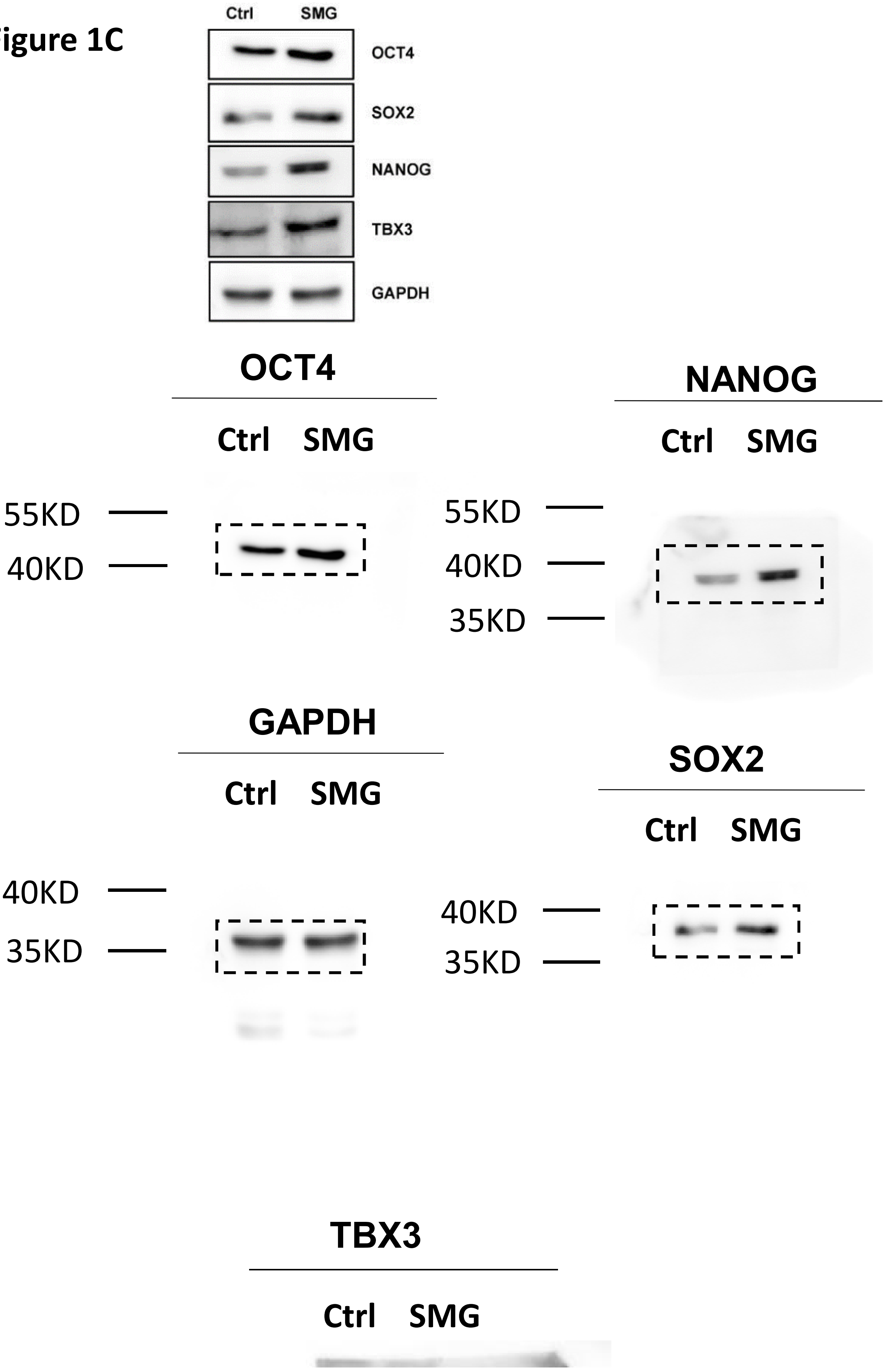

Ctrl
SMG
OCT4
SOX2
NANOG
TBX3
GAPDH
OCT4
Ctrl SMG
55KD
40KD
NANOG
Ctrl SMG
55KD
40KD
35KD
GAPDH
Ctrl SMG
40KD
35KD
SOX2
Ctrl SMG
40KD
35KD
TBX3
Ctrl SMG

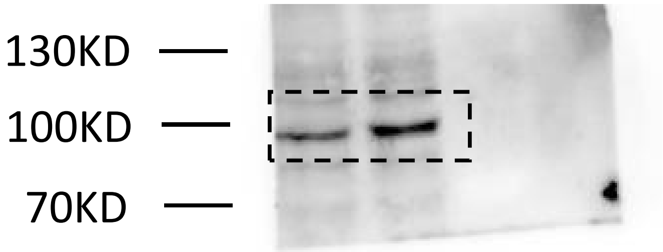
130KD
100KD
70KD

Figure 2F

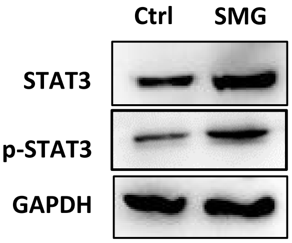
Ctrl
SMG
STAT3
p-STAT3
GAPDH

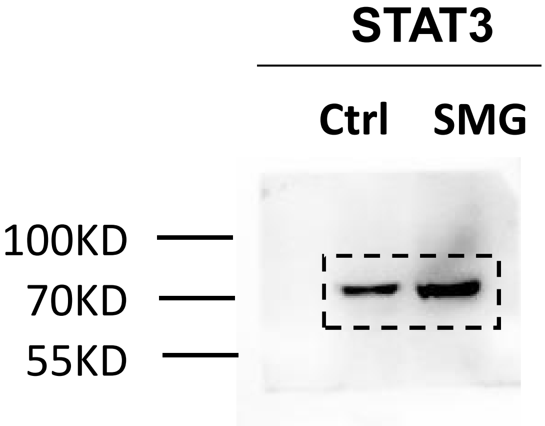
STAT3
Ctrl
SMG
100KD
70KD
55KD

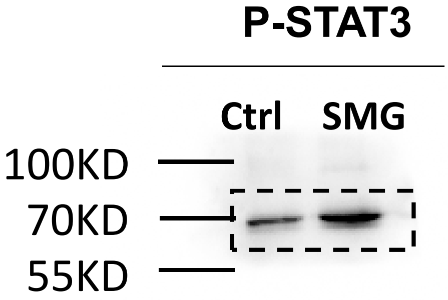
P-STAT3
Ctrl
SMG
100KD
70KD
55KD

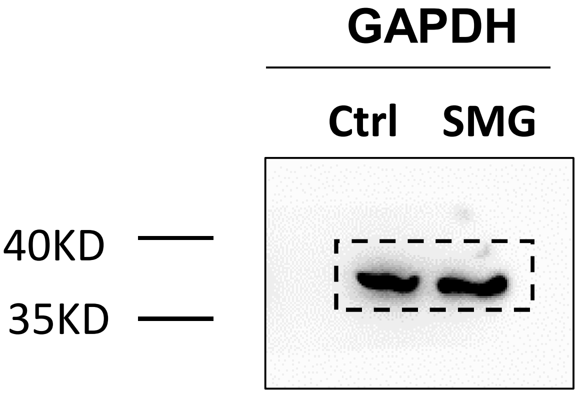
GAPDH
Ctrl
SMG
40KD
35KD

Figure 2G

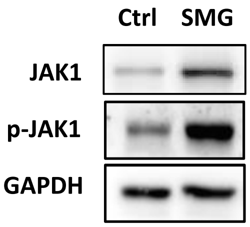

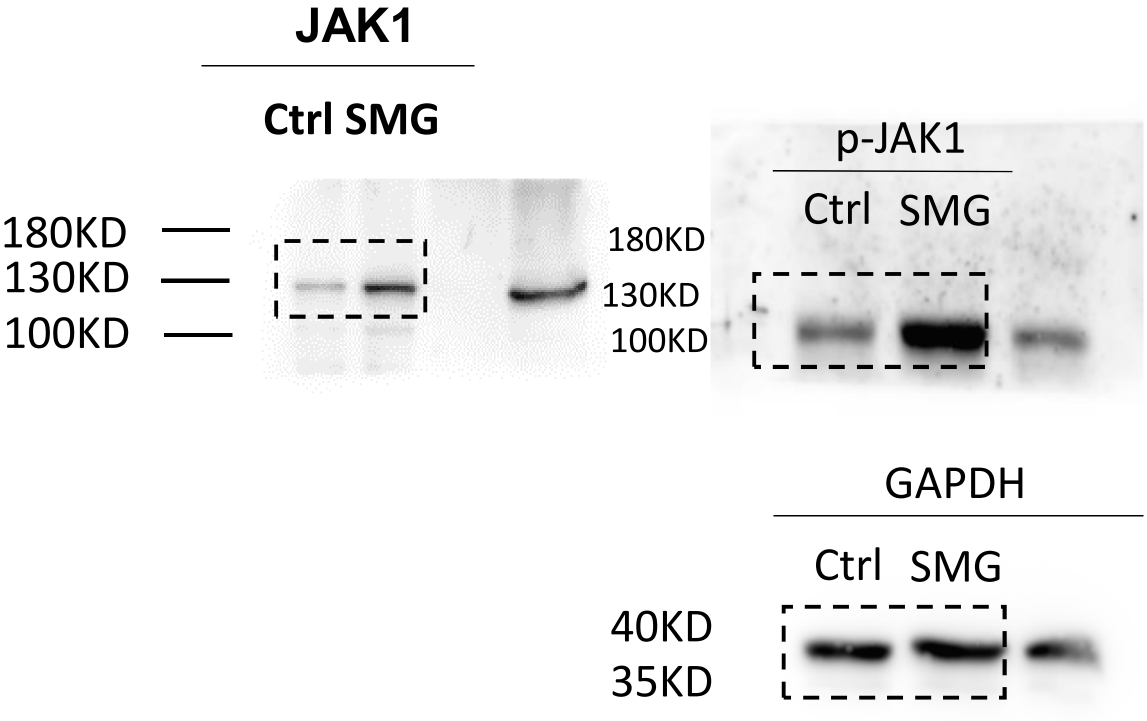

Figure 2H

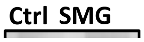

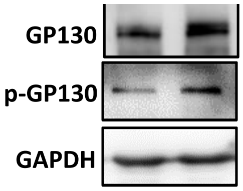

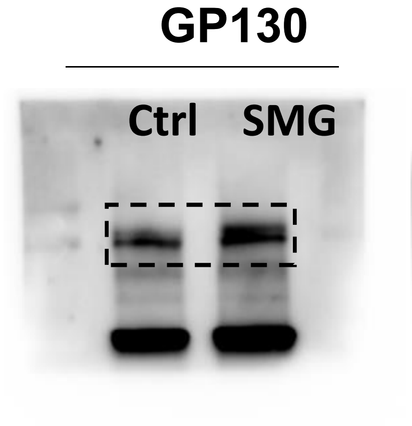

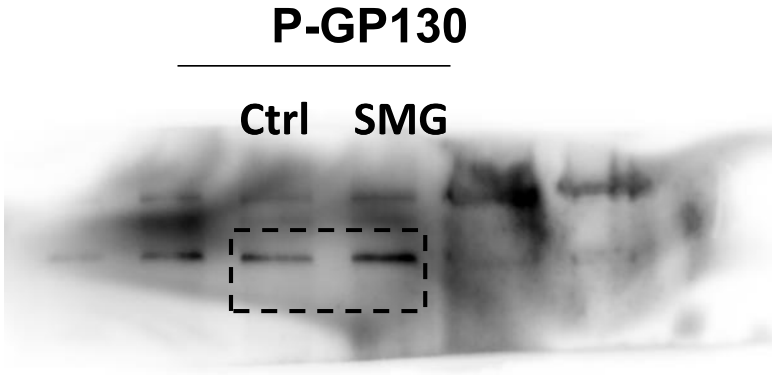

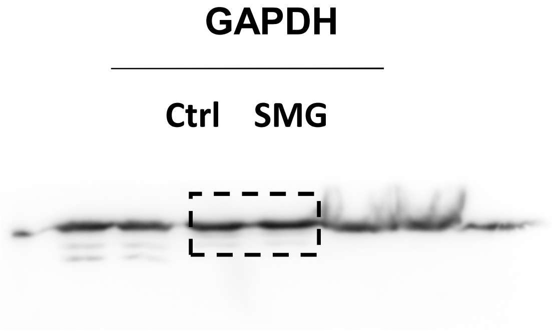

Figure S2E

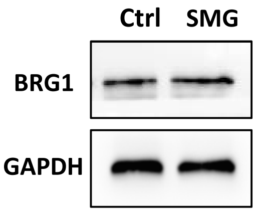

BRG1

GAPDH

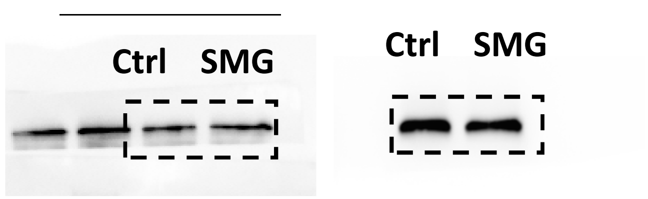
Ctrl SMG
Ctrl SMG

Figure S2H

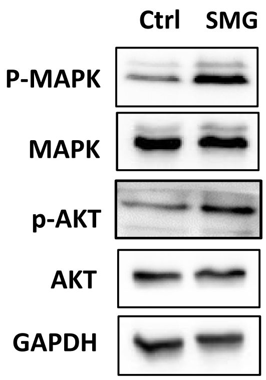
Ctrl SMG
P-MAPK
MAPK
p-AKT
AKT
GAPDH

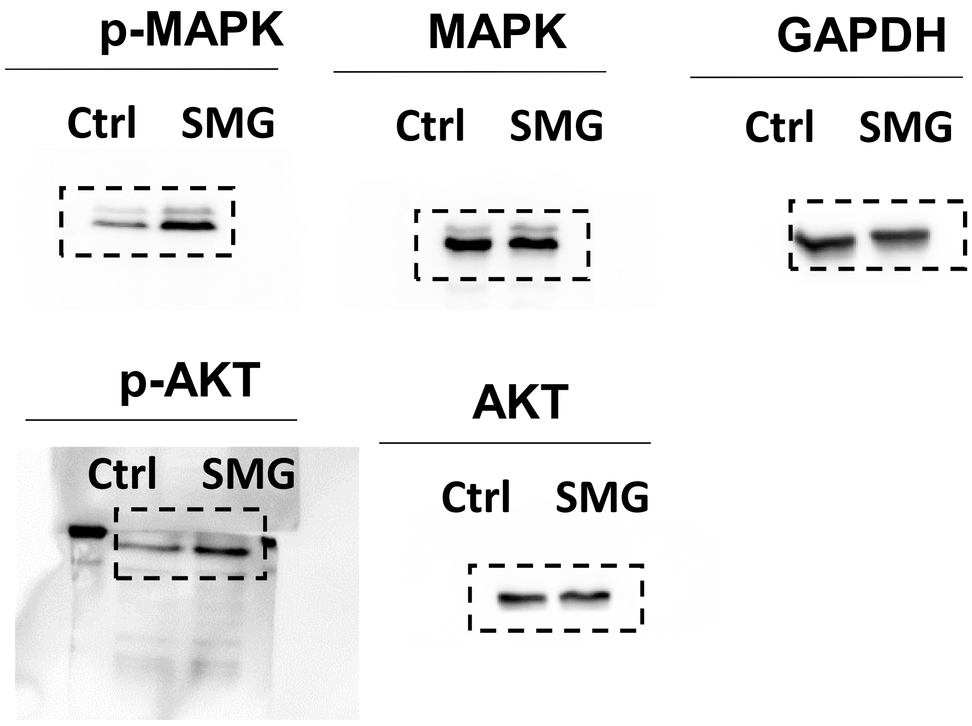
p-MAPK
Ctrl SMG
MAPK
Ctrl SMG
GAPDH
Ctrl SMG
p-AKT
Ctrl SMG
AKT
Ctrl SMG

Figure3D

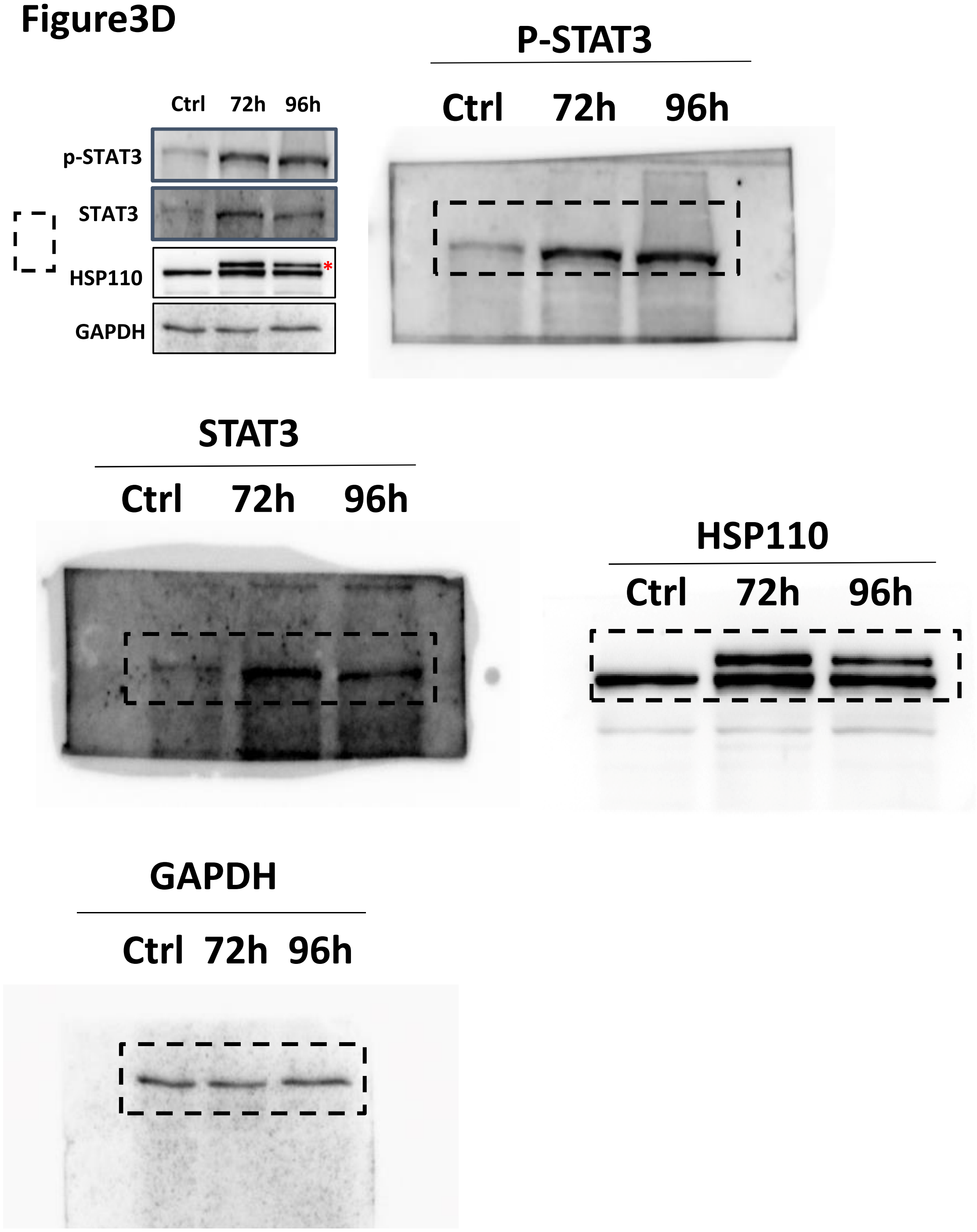

Ctrl 72h 96h
p-STAT3
STAT3
HSP110
*
GAPDH
P-STAT3
Ctrl 72h 96h
STAT3
Ctrl 72h 96h
HSP110
Ctrl 72h 96h
GAPDH
Ctrl 72h 96h

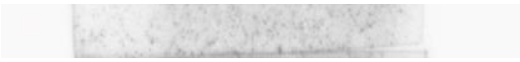

Figure S3B

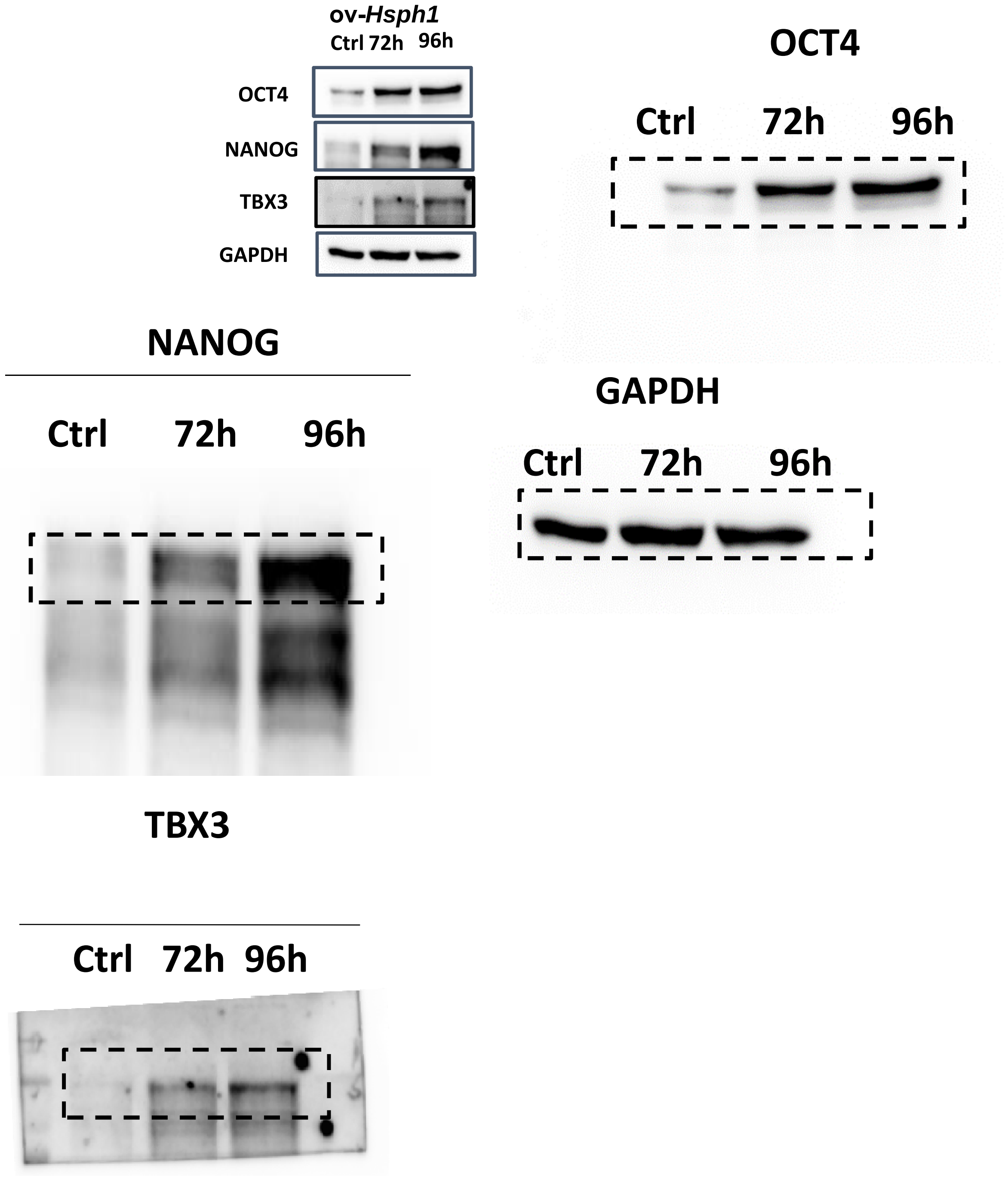
ov-*Hsph1*
Ctrl 72h 96h
OCT4
NANOG
TBX3
GAPDH
OCT4
Ctrl 72h 96h
NANOG
Ctrl 72h 96h
GAPDH
Ctrl 72h 96h
TBX3
Ctrl 72h 96h

Figure S3F

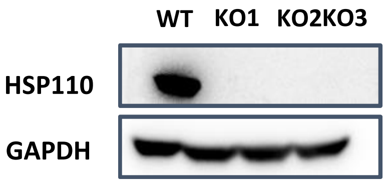

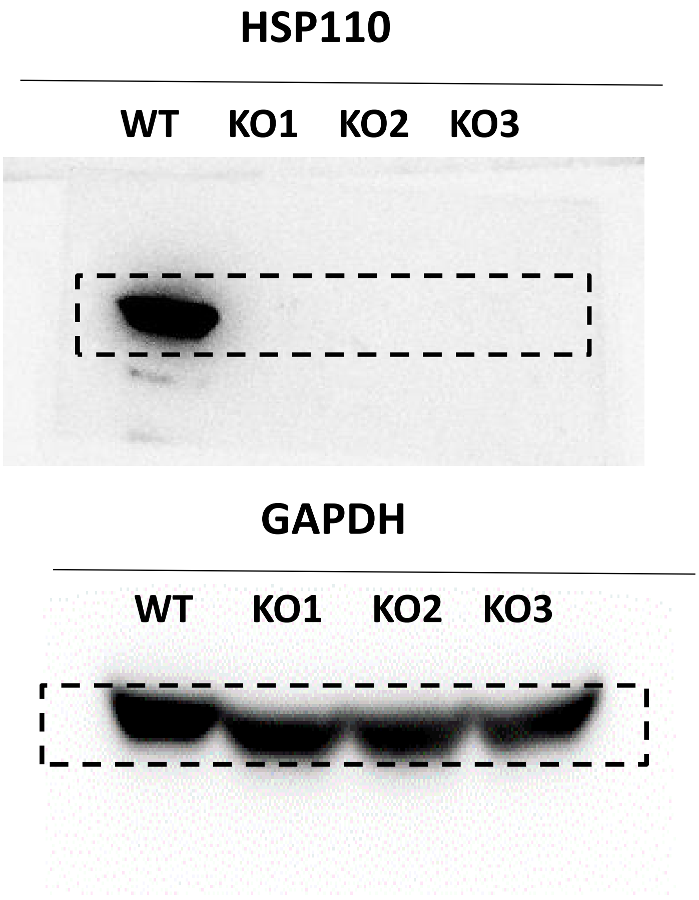

Figure S3G

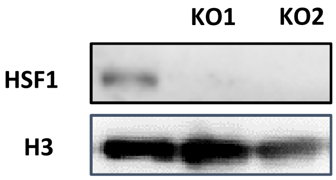

HSF1

Ctrl KO1 KO2

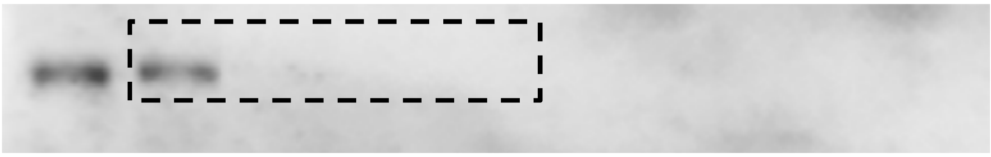

GAPDH

Ctrl KO1 KO2

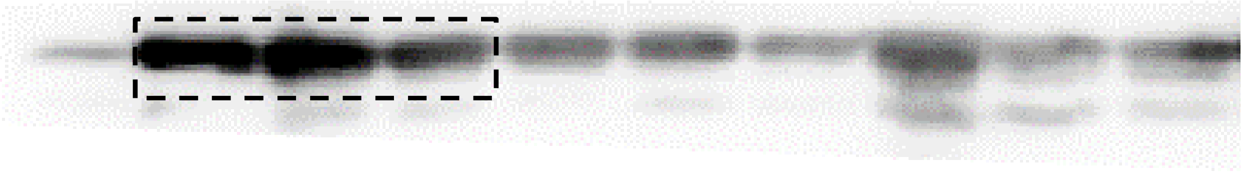

Figure 4G

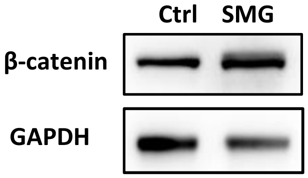

β-CATENIN

Ctrl SMG

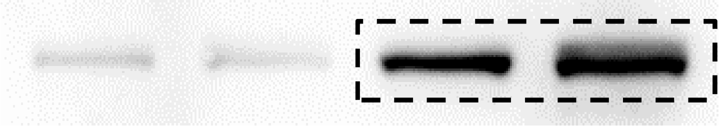

GAPDH

Ctrl SMG

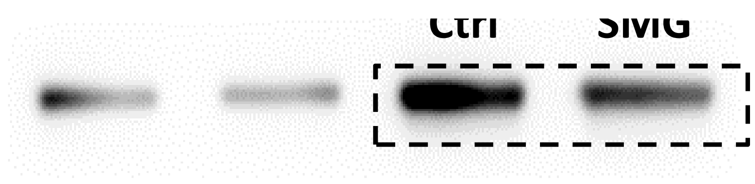
Ctrl
SMG
